# The anticancer potential of the CLK kinases inhibitors 1C8 and GPS167 revealed by their impact on the epithelial-mesenchymal transition and the antiviral immune response

**DOI:** 10.18632/oncotarget.28585

**Published:** 2024-05-16

**Authors:** Lulzim Shkreta, Johanne Toutant, Aurélie Delannoy, David Durantel, Anna Salvetti, Sophie Ehresmann, Martin Sauvageau, Julien A. Delbrouck, Alice Gravel-Trudeau, Christian Comeau, Caroline Huard, Jasmin Coulombe-Huntington, Mike Tyers, David Grierson, Pierre-Luc Boudreault, Benoit Chabot

**Affiliations:** ^1^Department of Microbiology and Infectious Diseases, Faculty of Medicine and Health Sciences, Université de Sherbrooke, Sherbrooke, QC, Canada; ^2^International Center for Infectiology Research (CIRI), INSERM U1111, CNRS UMR5308, Université de Lyon (UCBL1), Lyon, France; ^3^Institut de recherches cliniques de Montréal, Montréal, QC, Canada; ^4^Department of Pharmacology, Faculty of Medicine and Health Sciences, Université de Sherbrooke and Institut de Pharmacologie, Sherbrooke, QC, Canada; ^5^Institute for Research in Immunology and Cancer, Université de Montréal, Montréal, QC, Canada; ^6^Faculty of Pharmaceutical Sciences, University of British Columbia, Vancouver, BC, Canada

**Keywords:** CLK kinases inhibitors, EMT, antiviral immune response, microtubules, metastasis

## Abstract

The diheteroarylamide-based compound 1C8 and the aminothiazole carboxamide-related compound GPS167 inhibit the CLK kinases, and affect the proliferation of a broad range of cancer cell lines. A chemogenomic screen previously performed with GPS167 revealed that the depletion of components associated with mitotic spindle assembly altered sensitivity to GPS167. Here, a similar screen performed with 1C8 also established the impact of components involved in mitotic spindle assembly. Accordingly, transcriptome analyses of cells treated with 1C8 and GPS167 indicated that the expression and RNA splicing of transcripts encoding mitotic spindle assembly components were affected. The functional relevance of the microtubule connection was confirmed by showing that subtoxic concentrations of drugs affecting mitotic spindle assembly increased sensitivity to GPS167. 1C8 and GPS167 impacted the expression and splicing of transcripts in pathways relevant to tumor progression, including MYC targets and the epithelial mesenchymal transition (EMT). Finally, 1C8 and GPS167 altered the expression and alternative splicing of transcripts involved in the antiviral immune response. Consistent with this observation, depleting the double-stranded RNA sensor DHX33 suppressed GPS167-mediated cytotoxicity on HCT116 cells. Our study uncovered molecular mechanisms through which 1C8 and GPS167 affect cancer cell proliferation as well as processes critical for metastasis.

## INTRODUCTION

The CDC2-like kinases (CLKs) form a family of four kinases (CLK1-CLK4) that phosphorylate splicing regulatory SR proteins [1]. While all SR proteins are phosphorylated by the CLKs, SRSF10 may act as a sensor because it is a weaker substrate whose function is preferentially affected by small drops in CLK activity [2–6]. CLK1 and CLK2 are upregulated in various cancers including breast, colorectal, prostate, and glioblastoma [7]. As their role in cancer progression is increasingly recognized, the CLKs are emerging as promising therapeutic targets. Inhibition of CLKs suppresses cancer cell growth and induces apoptosis by modulating alternative splicing of genes involved in cell cycle, growth, and survival [7]. The CLK inhibitor TG003 alters splicing of cancer-associated genes and induces apoptosis and G2/M cell cycle arrest in prostate cancer cells [7]. The pan-CLK inhibitor SM08502 displays anti-tumor activity in gastrointestinal cancer models [8], while the potent CLK2 inhibitor T-025 demonstrates anti-tumor efficacy in an allograft model of MYC-driven breast cancer [9]. Combination of CLK inhibitors with Bcl-xL/Bcl-2 inhibitors synergistically induces apoptosis in cancer cells by modulating splicing and expression of anti-apoptotic proteins [10]. Inhibiting CLKs, either alone or in combination with other targeted therapies, may therefore represent a promising approach to selectively induce apoptosis and suppress tumor growth by modulating splicing of cancer-relevant genes.

1C8 and GPS167 are also inhibitors of CLK kinases. 1C8 was initially reported as an anti-HIV agent, but it also affects viral RNA maturation of HBV transcripts [3, 11, 12]. 1C8 reprograms the SRSF10-dependent alternative splicing of *BCLAF1* and *SREK1* in colorectal and hepatocellular carcinoma cell lines, respectively [3, 13]. Relative to 1C8, GPS167 more efficiently shifts *BCLAF1* splicing towards the non-oncogenic *BCLAF1-S* variant, and is cytotoxic to colorectal cancer cell lines and organoids [2]. While the GPS167-mediated toxicity is p53- and SRSF10-dependent, the depletion of SRSF10, which completely shifts *BCLAF1* splicing from the tumorigenic *BCLAF1-L* to the non-tumorigenic *BCLAF1-S* variant, does not impact the proliferation of HCT116 cells in culture [2]. Thus, given that SRSF10 and *BCLAF1-L* are overexpressed in high grade tumors [14], their functions may be more important for malignant progression. Compounds like 1C8 and GPS167 that inhibit or modulate the activity of SRSF10 could therefore become useful therapeutics against aggressive forms of cancer. Notably, GPS167 influences cell proliferation even in the absence of SRSF10 [2]. Thus, further investigation into the molecular pathways impacted by these compounds may unveil additional targets that could synergize with combinatorial therapeutic interventions, thereby enhancing their efficacy and broadening their therapeutic potential.

Given that both 1C8 and GPS167 affect SRSF10 phosphorylation, we hypothesized that investigating the effects of these two compounds would uncover shared pathways relevant to cancer progression. In this study, we employed kinase assays, a chemogenomic screen, and transcriptome analyses to elucidate the molecular pathways modulated by 1C8 and GPS167. Our investigation reveals a convergence of impacts for 1C8 and GPS167 on the epithelial-mesenchymal transition (EMT). Furthermore, both compounds induce a dsRNA-mediated antiviral immune response. Collectively, the properties exhibited by 1C8 and GPS167 suggest their potential utility in inhibiting tumor growth and metastasis.

## RESULTS

### 1C8 and GPS167 affect the proliferation of cancer cell lines

The anticancer potential of 1C8 was first hinted by its ability to shift SRSF10-dependent *BCLAF1* splicing towards the non-tumorigenic splice variant *BCLAF1-S* [3]. 1C8 also affects SRSF10-dependent *SREK1* alternative splicing in a hepatocellular carcinoma cell line, and inhibits the growth of Hep3B cells [13]. We used the NCI-60 tumor cell lines screening service at NIH/NCI to test the impact of 1C8 on the proliferation of a panel of cell lines from different types of cancer [15]. The results indicate that 1C8 exhibits a broad range of anti-proliferative effects (Supplementary Figure 1). Most cell lines proliferated at a reduced rate, with the highest impact, possibly due to cell death, on renal cancer cell lines (e.g., CAKI-1), the ovarian cancer cell lines SKOV-3 and IGROV1, the melanoma cell line UACC-62, as well as the breast cancer line MCF-7. The proliferation of cell lines derived from tumors of the central nervous system were among the least affected by 1C8.

We have shown previously that GPS167 promotes p53-dependent apoptosis in HCT116 cells [2]. GPS167 also affects cell proliferation and is cytotoxic for several other colorectal cancer cell lines and colorectal organoids, with no impact on normal colonocyte lines and organoids at the concentrations tested [2]. The NCI-60 screen also revealed an impact on the proliferation of cell lines from different types of cancers, including melanoma, leukemia, breast, ovarian and renal cancer [2].

### Impact of 1C8 and GPS167 on kinases

1C8 and GPS167 inhibit the activity of kinases of the CLK family [2, 5, 16]. A kinome screen performed with 1C8 on 421 kinases revealed CLK1, CLK2 and CLK4 as most strongly inhibited (less than 5% remaining activity at 10 μM 1C8; Supplementary Table 1). The SR protein kinases DYRK1A and DYRK1B (but not DYRK2 and DYRK3) were also strongly affected (approximately 10% remaining activity). The HIPK2 and the SRPK kinases were moderately and minimally affected, respectively. Non-SR protein-related kinases whose activities were below 20% of the initial activity with 10 μm of 1C8 included CSNK2 (aka CK2; 5% remaining activity), GCN2 (aka EIF2AK4; 8%), FLT3, FLT4, IRAK1, IRAK4, MER, NEK4 and TRB2 (Supplementary Table 1, Supplementary Figure 2).

A radiometric kinase assay indicated that GPS167 strongly impacts CLK1, CLK2 and CLK4, with a 3 to 5 fold lower sensitivity for DYRK1B and DYRK2 relative to CLK1 [16]. In contrast to 1C8, GPS167 did not significantly inhibit CSNK2 (Supplementary Figure 3).

### Impact on microtubule function

**Figure 1:**
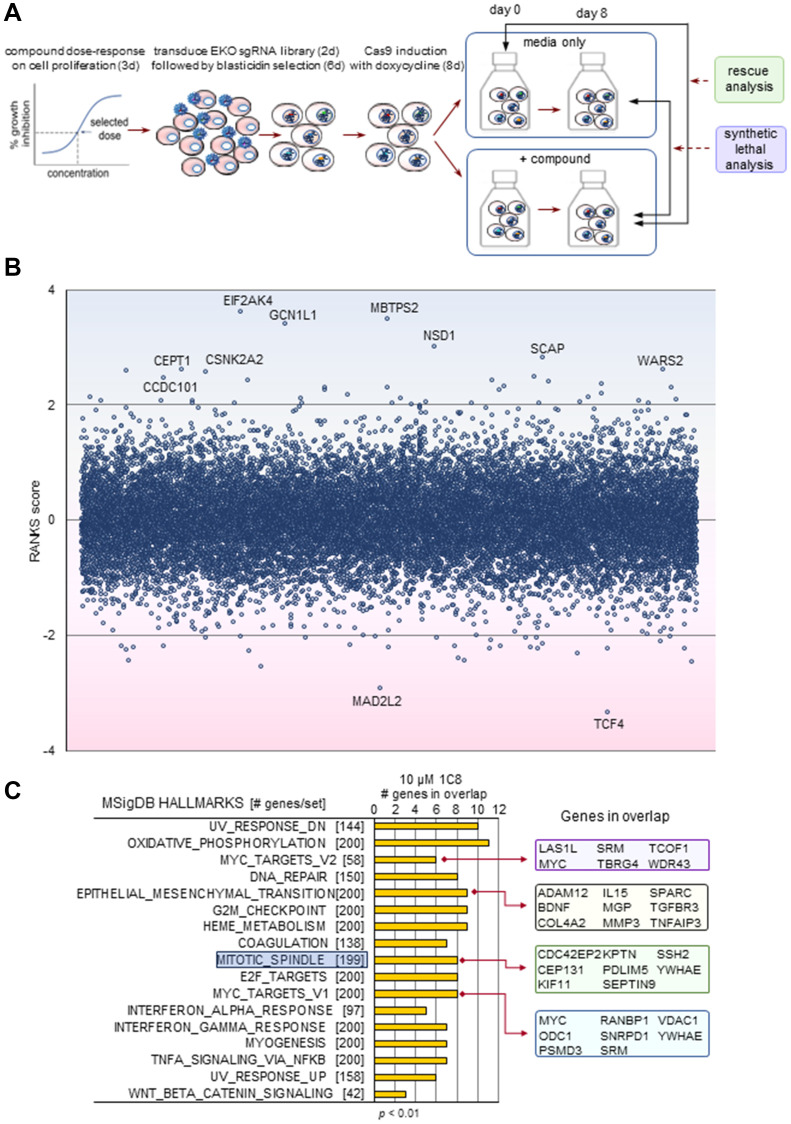
The CRISPR-based chemogenomic screen with 1C8. (**A**) Schematic overview of whole genome pooled CRISPR screens carried out with the NALM-6 cell line. (**B**) CRISPR screen RANKS scores for 1C8. Positive scores and blue shading indicate a drug-resistant (rescue) phenotype while negative scores and pink shading indicate a drug-sensitive (synthetic lethal) phenotype. Genes with scores greater than 1 or less than -1 are shown. Labelled genes indicate significant hits (FDR < 0.05). (**C**) The most significantly enriched Gene Ontology terms in the top 250 genes of the highest and 250 genes of the lowest RANK score were selected to identify subset of highest impact genes in the MSigDB hallmarks gene sets (GSEA).

To gain insight into the molecular genetic pathways, we carried out whole-genome CRISPR/Cas9 knockout screens using the Extended Knockout (EKO) library of over 275 000 single-guide RNAs (~10 guides per gene) and the B cell precursor leukemia cell line NALM-6, as described previously [2] (Figure 1A). A chemogenomic screen previously carried out with GPS167 in NALM-6 cells indicated that the top 10 of the top 50 synthetic lethal genes were related to the mitotic cell cycle checkpoint or chromosome segregation, suggesting mitotic spindle dysfunction [2]. Several top rescue hits were similarly linked to mitotic spindle assembly [2]. A transcriptome analysis based on a single RNA-Seq sample of HCT116 cells treated with GPS167 identified several genes in the mitotic spindle category experiencing differential mRNA expression and alternative splicing [2]. A Gene Set Enrichment Analysis (GSEA) [17] analysis identified *mitotic spindle* as a category significantly affected both in expression and splicing by GPS167 (Supplementary Figures 4 and 5).

**Figure 2:**
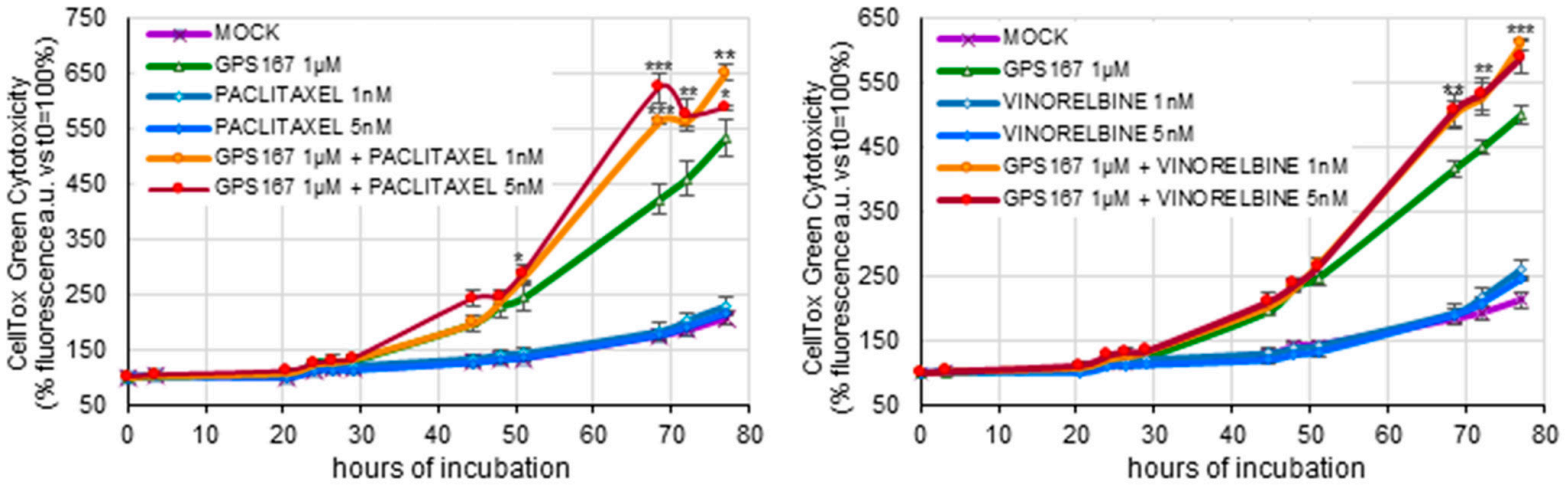
Impact of anti-microtubule drugs on GPS167-mediated cytotoxicity. The CellTox-Green assay was used to monitor cytotoxicity of treated and untreated HCT116 cells at different times using two concentrations of paclitaxel (left panel) or vinorelbine (right panel) in the absence or the presence of 1 μM GPS167. The statistical analysis for the combined treatments was performed using multiple *t*-test (GraphPad Prism software, version 10.2.2). ^*^ *p* < 0.05, ^**^ *p* < 0.01 and ^***^ *p* < 0.001. We noted a slight drop in apparent toxicity at the 72h time point with GPS167 and 5 nM paclitaxel. One possibility to explain this drop is that the extensive cell death associated with this treatment at that time and the later time point led to less efficient binding of the dye to more extensively degraded DNA, thereby underestimating cytotoxicity.

Given the potential impact of GPS167 on the function of microtubule components, we tested GPS167 on HCT116 cells in combination with two drugs that antagonize mitotic spindle assembly (Figure 2). Non-toxic concentrations of the microtubule-stabilizing agent paclitaxel and the microtubule-destabilizing agent vinorelbine stimulated the cytotoxic response to GPS167. Thus, affecting microtubule function increased the sensitivity of cancer cells to GPS167. A similar synergy is known to occur when combinations of microtubule targeting drugs are used [18].

Performing the chemogenomic screen with 1C8 in NALM-6 cells yielded sets of hits that were different than with GPS167, possibly indicating different mechanisms of action (Figure 1B and Supplementary Table 2). A GSEA analysis of the top screen hits for 1C8 indicated top enrichment scores for components involved in DNA damage and oxidative phosphorylation (Figure 1C). *Mitotic spindle* also came up as a category significantly affected by 1C8. The knockout of *CSNK2A2* improved growth of NALM-6 cells in the presence of 1C8 (Figure 2). CSNK2A2 is a component of the mitotic spindle assembly checkpoint [19], and is the catalytic subunit of CSNK2 inhibited by 1C8 (Supplementary Table 1, Supplementary Figures 2 and 3). The chemogenomic screen also identified the loss of *MAD2L2* as synthetic lethal for 1C8. *MAD2L2* encodes a mitotic spindle assembly checkpoint protein. Notably, in fission yeast, MAD2p fails to localize to the kinetochore when CSNK2 is inhibited [20]. Since ectopic expression of MAD2p counteracts the impact of a CSNK2 mutant [20], this could explain why *MAD2L2* would be essential when 1C8 inhibits CSNK2 in NALM-6 cells.

A GSEA analysis of the expressed transcriptome of HeLa cells treated with 1C8 (using triplicate samples, as previously reported in Shkreta et al. 2017) identified *mitotic spindle* as one of the top categories (Supplementary Figure 6). As for transcripts whose alternative splicing was altered by 1C8, a GSEA analysis indicated that several mitotic spindle components were affected (Supplementary Figure 7).

### Impact on the epithelial-mesenchymal transition

**Figure 3:**
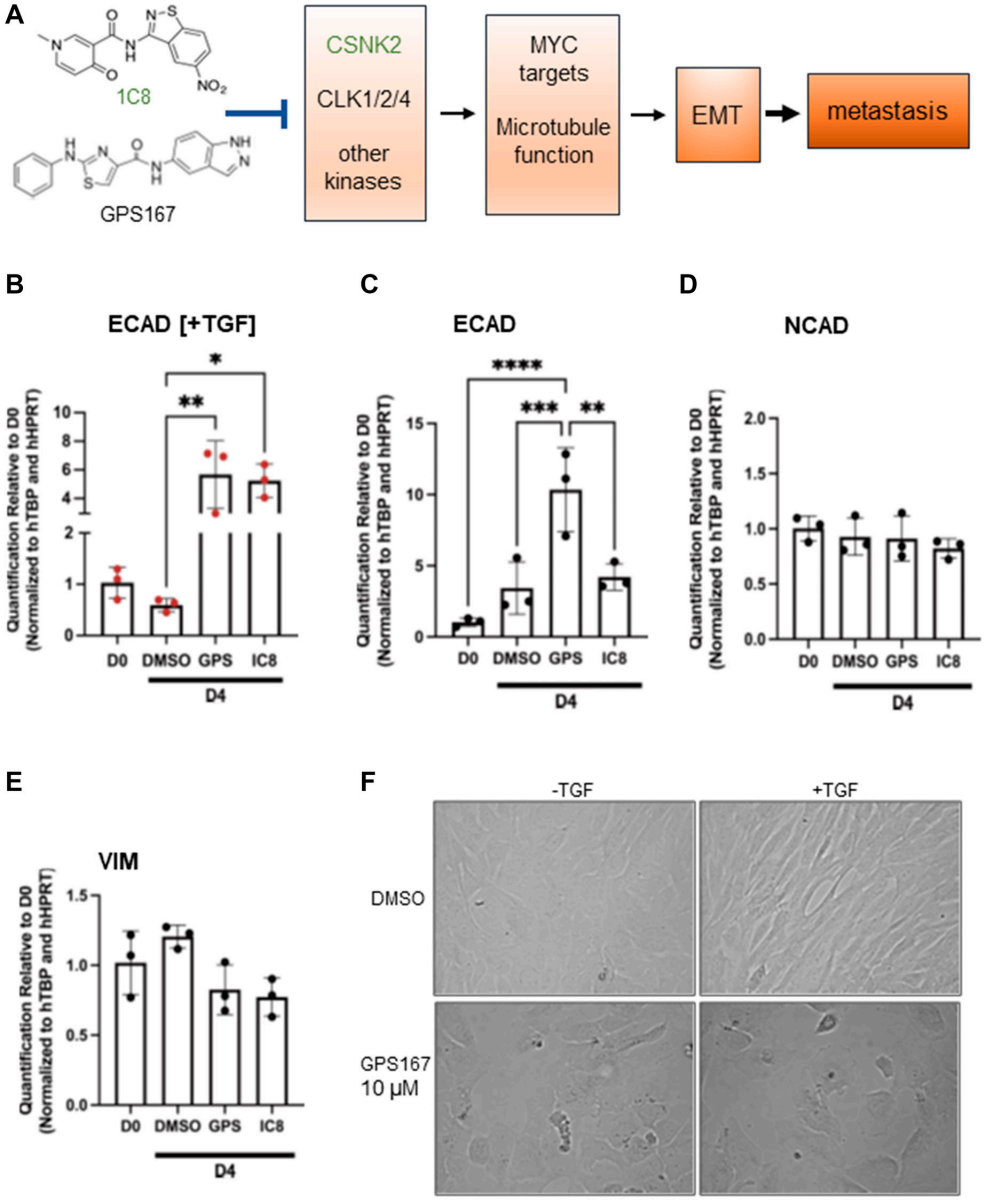
Impact of 1C8 and GPS167 on EMT. (**A**) The model illustrates our view of the mechanism of action of kinase inhibitors 1C8 and GPS167. The inhibition of CLK kinases 1/2/4 and possibly other kinases (only 1C8 affects CSNK2 activity) impacts the expression and alternative splicing of MYC target genes and genes involved in microtubule function. These changes would affect EMT, a process essential for metastasis. (**B**–**E**) qRT-PCR assays to monitor expression of epithelial and mesenchymal markers. Assays are performed on MCF10A cells induced for 24 hours with (B) or without (C–E) TGF-β. Statistical significance was calculated with Tukey’s multiple comparison test. (**F**) Microscope images of MCF10A cells treated for 4 days with DMSO or GPS167 and with or without TGF-β.

Consistent with the chemogenomic screens, which indicated that the knockout of several components linked to mitotic spindle impacted cell proliferation in the presence of 1C8 and GPS167, both compounds affected the expression and the alternative splicing of transcripts associated with microtubules and mitotic spindle function. While mitotic spindle components are important for cell growth, additional contributions to processes relevant to aggressive tumor progression are emerging. For example, the nucleolar and spindle-associated protein 1 (*NUSAP1*) promotes cell migration and invasion of prostate and colorectal cancer cells [21, 22]. Given the role of CSNK2 in microtubule function, it is also notable that CSNK2 activation is required for transforming growth factor β (TGF-β)-induced epithelial-mesenchymal transition (EMT) [23], a process that is intimately relevant to tumor invasion (Figure 3A).

Notably, EMT and pathways connected to EMT were top categories in our chemogenomic screens and transcriptome analyses. The MYC activator TCF4, which is part of the β-catenin complex that induces EMT and tumor invasion [24, 25], was a top synthetic lethal hit with both GPS167 and 1C8 (Figure 1B) [2]. MYC target genes make multiple contribution to metastasis, including functional associations with EMT, angiogenesis and invasion [26]. MYC target genes were top categories of hits in the chemogenomic screen performed with 1C8 (Figure 1C). Likewise, EMT, TGF-β signaling, Notch signaling and MYC targets were among the top categories whose expression and alternative splicing were impacted by 1C8 in HeLa cells (Supplementary Figures 6 and 7). An analysis using the EMTome database [27] confirmed a broad disruption in the expression of EMT-relevant genes, with 9 genes overexpressed more than 3-fold with 5 μM or more of 1C8 (*ECM2, ALPK2, EDN1, ACTA2, MSC-AS1, FOXQ1, NECTIN4, NNMT* and *THBS1*), and 5 genes repressed by more than 2.5-fold (*TNC, SLIT2, JUN, ANGPTL4* and *CA2*) (Supplementary Figure 8A). Expression of epithelial-specific *CLDN1* was stimulated, while that of mesenchymal-specific *ACTA2* and *FAP* were respectively stimulated and repressed by 1C8 (Supplementary Figure 8B). Likewise, several changes in the alternative splicing of transcripts encoding mesenchymal markers were noted (Supplementary Figure 8C). Thus, 1C8 affects the expression and splicing of genes intimately associated with EMT.

GPS167 also affected the expression and splicing of genes relevant to EMT (e.g., Notch and TGF-β signaling, MYC targets; Supplementary Figures 4 and 5). Disruption in the expression of several genes listed in the EMTome, and in signature genes for the epithelial and the mesenchymal states were observed (Supplementary Figure 9A, 9B). We previously identified an alternative splicing change in *FLNB* that favors a variant promoting the mesenchymal state in human breast cancer [2]. Another GPS167-mediated splicing disruption occurred in transcripts encoding CD44 (Supplementary Figure 9C), which plays an important role in metastasis [28, 29].

To confirm experimentally the above link with EMT, we tested the impact of GPS167 and 1C8 in a TGF-β-induced EMT assay using MCF10A cells. ECAD is an epithelial marker whose loss of expression disrupts cell adhesion to increase cell motility and EMT in cancer cells [30]. TGF-β normally elicits a small drop in the level of ECAD, whereas the presence of GPS167 and 1C8 stimulated ECAD expression in these conditions (Figure 3B). For cells not treated with TGF-β, GPS167 also promoted expression of ECAD (Figure 3C). On the other hand, the expression of mesenchymal markers NCAD and vimentin was not affected by the compounds (Figure 3D, 3E). Consistent with an impact on EMT, GPS167 prevented the change in cell morphology normally associated with EMT from the cobblestone-like appearance characteristic of epithelial cells to a more elongated, spindle-shaped morphology (Figure 3F). Overall, these results suggest that 1C8 and GPS167 interfere with the transition from the epithelial to the mesenchymal state.

The above analysis indicates that 1C8 and GPS167 impact the expression and splicing of genes involved in tumor development and malignant progression. In addition to common pathways being involved, we identified 13 genes whose expression, and 20 transcripts whose splicing were similarly affected by 1C8 and GPS167 (>2-fold change and > 15% change in PSI, respectively) (Supplementary Figure 10A, 10B). In shared genes having experienced a change in expression, *TP53I3, SESN2, XPC, BAX, SFN, TP53INP1* and *SAT1* formed a group united by their connections to p53 regulation. In addition, we noted a strong 1C8-mediated impact in the alternative splicing of the EMT-relevant *ITGA6* transcript (ΔPSI = 48%) that was also affected (ΔPSI = 11%) by GPS167 [2] (Supplementary Figure 10C). Shared splicing disruptions were identified in units displaying more complex alternative splicing profiles. For instance, both 1C8 and GPS167 promoted a 20% drop in the production of the *QK1-7B* splice variant (Supplementary Figure 10D). *QK1* splice variants have recently been shown to influence migration and EMT associated properties [31, 32]. Finally, 1C8 and GPS167 promoted several splicing changes in the N-terminal regulatory portion of transcripts encoding CTNND1 (Catenin-δ1) (Supplementary Figure 10E), a protein that binds cadherins and plays a critical role in EMT and metastasis [33, 34].

### 1C8 and GPS167 elicit an antiviral response

An additional feature of our GSEA analysis of the impact of 1C8 and GPS167 was alterations in immune signaling pathways. GPS167 downregulated the expression of IFNα-responsive genes, while IFNγ-responsive genes were upregulated, as were TNFα signaling genes and inflammatory response genes (Supplementary Figure 4). Likewise, the alternative splicing of transcripts involved in TNFα signaling, inflammatory and IFNα response was affected in HCT116 cells treated with GPS167 (Supplementary Figure 5). The IFNα/IFNγ pathways, and TNFα signaling through nuclear factor kB (NFKB) were among the most significant enriched categories in cells treated with 1C8 (Supplementary Figure 6). 1C8 also impacted the alternative splicing of transcripts involved in the IFNα and IFNγ response, as well as in TNFα signaling (Supplementary Figure 7).

**Figure 4:**
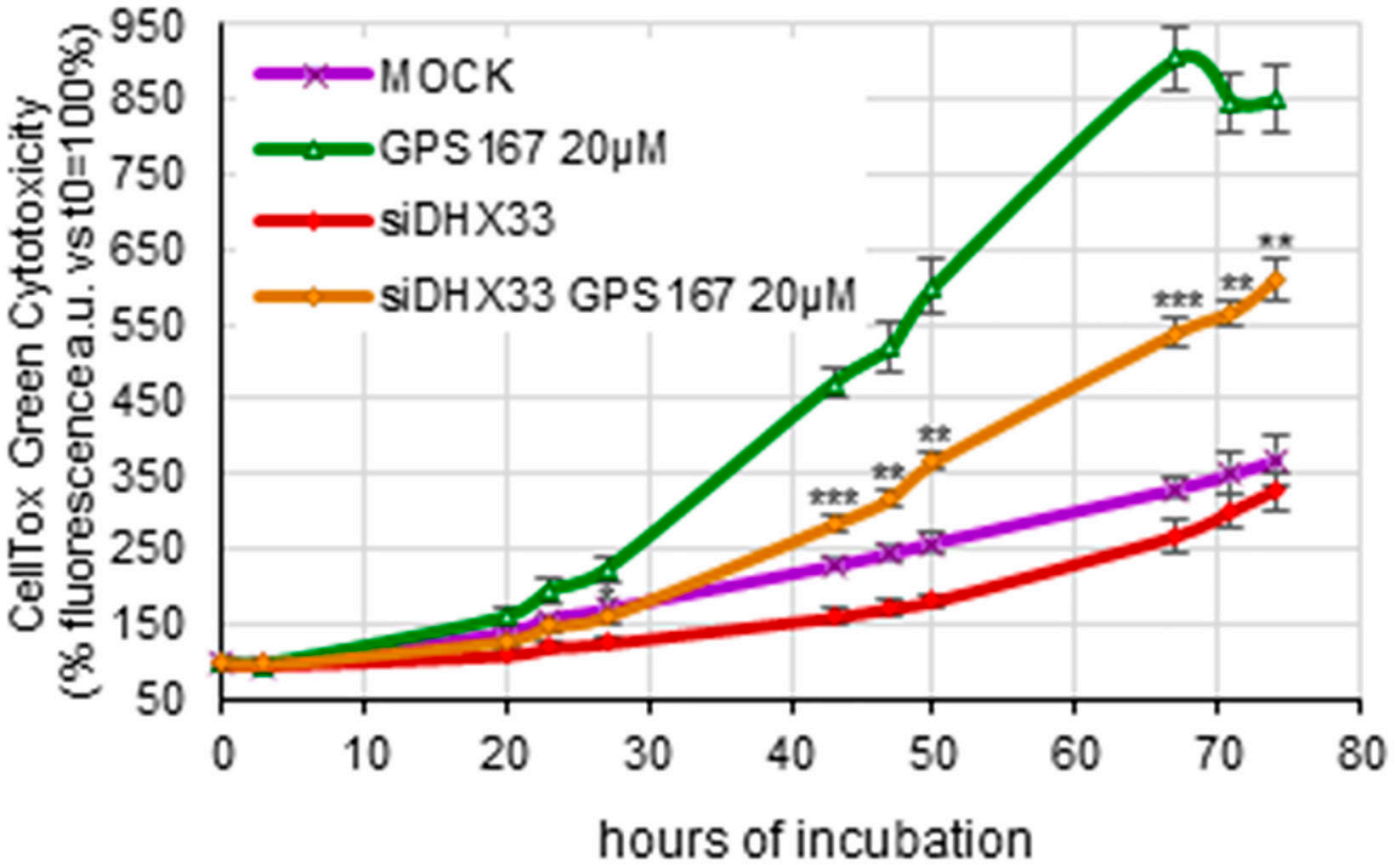
Depletion of DHX33 antagonizes GPS167-mediated cytotoxicity. The CellTox-Green assay was used to monitor cytotoxicity of HCT116 cells depleted or not of DHX33 at different times and with or without 20 μM GPS167. For each time-point, the cytotoxicity of DHX33-depleted cells versus mock-treated cells, both treated with 20 μM GPS167, was compared using multiple *t*-test analysis (GraphPad Prism software, version 10.2.2). ^*^ *p* < 0.05, ^**^ *p* < 0.01 and ^***^ *p* < 0.001.

Overexpression of type I IFNs (which include IFNα but not IFNγ) and NFKB-responsive genes was recently associated with the accumulation of intron-retained mRNAs that induce cytoplasmic dsRNA-sensing and signaling pathways that in turn trigger an antiviral immune transcriptional response [35]. Our analysis of the splicing impact of 1C8 indicates an accumulation of dozens of intron-retention events, while others were repressed (Supplementary Figure 11). dsRNA sensors such as DHX33 play a crucial role in activating the mitochondrial antiviral signaling protein MAVS to induce transcriptional changes and extrinsic apoptosis [35, 36]. We therefore assessed the role of the dsRNA sensor DHX33 in mediating the cytotoxic impact of GPS167. The siRNA-mediated knockdown of DHX33 suppressed the cytotoxic effect of GPS167 (Figure 4), indicating that GPS167 triggers dsRNA-sensing pathways that promote cell death.

## DISCUSSION

1C8 and GPS167 inhibit the activity of CLK kinases, which in turn affect the phosphorylation and activity of SRSF10. While SRSF10 is overexpressed in high grade human colorectal tumors to promote production of the tumorigenic *BCLAF-L* splice variant [14], the genetic depletion of SRSF10 has little impact on the proliferation of HCT116 cells in culture [2]. The CLK-SRSF10-BCLAF1 axis may therefore make more important contributions to processes associated with aggressive cancer such as cell migration and invasion. Consistent with this view, BCLAF1 has been implicated in angiogenesis, and both BCLAF1 and SRSF10 facilitate metastasis of hepatocellular carcinoma cells [37, 38]. SRSF10 also contributes to the development of gliomas [39], and controls the alternative splicing of *WTAP* [2], which is involved in the migration and invasion of cholangiocarcinoma cells [40].

Although 1C8 and GPS167 target CLK kinases, they also affect the activity of non-CLK kinases. The fact that the chemogenomic screen led to distinctive sets of hits for 1C8 and GPS167 suggests that a subset of kinases may be differentially affected by 1C8 and GPS167. One such case is CSNK2, which is inhibited by 1C8 but not GPS167. Thus, a mixture of CLK-associated and non-CLK mediated events triggered by 1C8 and GPS167 may contribute to their anti-cancer potential.

Our analysis identified several pathways affected by 1C8 and GPS167 that are relevant to aggressive stages of cancer. One pathway revealed by a chemogenomic screen is mitotic spindle assembly, a process that requires the nucleation of microtubules. 1C8 and GPS167 also affected the expression and the alternative splicing of transcripts involved in microtubule function. Consistent with a link between GPS167 and microtubules, subtoxic concentrations of anticancer drugs known to affect microtubule function (vinorelbine and paclitaxel) increased the cytotoxicity of GPS167. We showed previously that GPS167 affected the alternative splicing of the microtubule components *NUMA1* and the SRSF10-regulated *SLK1* in HCT116 cells [2]. The SLK1 kinase controls motility in breast cancer cells [41]. Notably, microtubules have been increasingly associated with metastasis [42]. For example, microtubule disruption reduced metastasis more than cell proliferation (Thompson et al. 2022). While it remains unclear exactly how inhibiting CLK kinases affects microtubule function, 1C8 also inhibits CSNK2 (casein kinase 2), a microtubule component that functions as part of the spindle assembly checkpoint [19]. Moreover, the knockdown of its catalytic component CSNK2A2 rescued proliferation in the chemogenomic screen with 1C8. Consistent with the involvement of microtubules and CSNK2 in late stages of cancer, overexpression of CSNK2A2 is associated with poor prognosis in ovarian cancer [43], while its inhibition reduces tumor growth and metastatic colonization [44]. CSNK2 is overexpressed in breast cancer, and two CSNK2 inhibitors are in clinical trials as anticancer drugs [45]. The sensitivity of renal cancer cell lines to 1C8 may also be related to CSNK2A2, which is a target in renal cancer [45].

Two cancer hallmarks strongly impacted by 1C8 and GPS167 based on the chemogenomic screen and expression/splicing analyses were MYC targets and EMT. MYC is highly relevant to EMT, a process that contributes to various aspects of tumor progression, including cell motility and metastasis [26, 46–48]. *MDM4*, whose splicing was previously shown to be regulated by SRSF10, and affected by GPS167 [2], may also be important for metastasis since its knockdown reduces circulating tumor cells in triple-negative breast cancer without affecting proliferation [49]. The alternative splicing of other EMT-relevant transcripts affected by the compounds may occur independently of SRSF10, as we have shown previously for *FLNB*, *MDM2* and *CD44* [2]. We further observed that in epithelial MCF-7 cells treated with TGF-β to elicit EMT, the expression of the epithelial marker ECAM was stimulated by 1C8 and GPS167, suggesting that the transition to the mesenchymal state may be impaired. This interference by 1C8 could be mediated at least in part by CSNK2 inhibition, which is required for TGF-β-induced EMT [23].

We have shown previously that p53 is required to mediate the cytotoxic effect of GPS167 [2], raising the possibility that the cytotoxic impact of the compounds on cancer cells may be lost when p53 is mutated during cancer progression. Notably, approximately 60% of colorectal cancers have a mutated p53 gene [50]. However, the loss of p53 has limited impact on the invasion process, with some p53 mutations eliciting a pro-metastatic phenotype, while others promote metastasis when combined with KRAS activation and TGF-β suppression [51–54]. Moreover, the loss of p53 or missense-type mutations in p53 induce NF-κβ, which may stimulate progression to EMT [50]. Thus, the EMT disrupting activity of 1C8 and GPS167 may become even more relevant in p53-mutated cancers. Furthermore, therapeutic interventions often promote EMT. As EMT represents a converging pathway for genes affected by 1C8 and GPS167, these compounds may therefore have potential to prevent cancer cells from becoming more invasive.

1C8 and GPS167 also perturbed the expression of transcripts involved in immune signaling pathways, including the IFNα response, the inflammatory response and TNFα signaling via NF-κβ. It was recently observed that RNA processing defects elicited by anti-splicing drugs lead to intron-derived dsRNA accumulation that when coupled to MYC overexpression induces antiviral immune signaling in tumors [35]. Notably, CLK1/4 activity has been reported to control intron retention events in human cells [55]. Thus, CLK kinases inhibition by 1C8 and GPS167 may lead to RNA mis-splicing events that, in combination with the overexpression of several MYC targets, may activate antiviral immune signaling. Consistent with this view, knocking down the dsRNA sensor DHX33 made GPS167 less cytotoxic for HCT116 cells, suggesting that GPS167-mediated RNA splicing aberrations provoke the formation of dsRNAs that eventually lead to cell death. Thus, 1C8 and GPS167, through antiviral signaling, may stimulate extracellular apoptosis. As for an impact on immune signaling, SRSF10 has recently been reported to inhibit the IFNα/IFNγ signaling pathway and suppress CD8^+^T cell infiltration, while its knockdown enhanced the anti-PD-L1-mediated anti-tumor activity [38]. In addition to CLK kinases-mediated events, the inhibition of GCN2 kinase by 1C8 may also help elicit the antiviral response [56].

Our analysis therefore identified several pathways affected by 1C8 and GPS167 that are relevant to aggressive stages of cancer. In addition to those discussed above, pathways including apoptosis, hypoxia, K-RAS signaling, DNA repair, and E2F targets are also impacted by both compounds. While we have shown that GPS167 elicits p53-dependent apoptosis [2], further investigation is warranted to comprehensively elucidate if and how these pathways contribute to the anti-cancer activity of our compounds.

In summary, we have characterized a pair of compounds that impact multiple processes that are relevant to cancer cell proliferation but also, and possibly more importantly, to metastasis, which is the main cause of cancer lethality. 1C8 and GPS167 shift splicing of *BCLAF1* to its non-tumorigenic variant, impact the expression and splicing of several categories of genes associated with EMT (i.e., microtubules, MYC targets, Notch and TGF-β signaling) and the viral immune response. Anticancer drugs with such multifaceted effects could have several advantages, as the simultaneous targeting of different pathways could be synergistic, in addition to potentially reducing the likelihood of developing drug resistance.

## MATERIALS AND METHODS

### Synthesis of GPS167 and 1C8

The synthesis of both compounds (1C8 and GPS167) followed previously established protocols with some modifications [12, 57]. Regarding GPS167 (Supplementary Figure 12), Ethyl 2-(phenylamino)thiazole-4-carboxylate (1) was produced by reacting ethyl 2-bromothiazole-4-carboxylate with aniline in a refluxing solution. Following hydrolysis to give compounds 2, it was coupled with 5-aminoindazole through HATU coupling and subsequently purified via reverse-phase preparative HPLC.

Compound 1C8 was synthesized through a four-step process beginning with 4-chloropyridine-3-carboxylic acid (Supplementary Figure 13). Initially, this compound underwent esterification to form methyl 4-methoxynicotinate 3, which was subsequently methylated using methyl iodide, resulting in methyl 1-methyl-4-oxo-1,4-dihydropyridine-3-carboxylate 4. This intermediate was then attached to 5-nitrobenzo[d]isothiazol-3-amine, synthesized separately by adding a TMS group to the amine on 3-amino-5-nitrobenzisothiazole. The final product 1C8 underwent purification using flash chromatography with a 5% MeOH/DCM mixture.

### CRISPR-based chemogenomic screens

The EKO pooled lentiviral library of 278 754 sgRNAs targeting 19 084 RefSeq genes, most with 10 guides per gene, 3 872 hypothetical ORFs and 20 852 alternatively spliced isoforms was introduced within a clone of the NALM-6 pre-B lymphocytic cell line with a doxycycline-inducible Cas9 was described previously [58]. NALM-6 cells at 200 000 cells per ml were exposed for a period of 3 days to a range of concentrations of 1C8, followed by assessment of cell proliferation by CellTiter-Glo assay (Promega) using a Biotek Synergy Neo multi-mode microplate reader. From these dose-response curves, we estimated that concentrations of 10 μM of 1C8 would inhibit growth sufficiently to observe growth rescue phenotypes in CRISPR screens while still allowing enough growth to also observe drug sensitivity phenotypes. The EKO library (kept at a minimum of 250 cells per sgRNA) was thawed and cultured in 10 % FBS RPMI supplemented with 2 mg/mL doxycycline for a period of 8 days to induce knockouts with dilutions to 400 000 cells per mL every 2 days. After 8 days, 70 × 10^6^ cells were spun at 1200 rpm for 5 min, washed with 1X PBS, pelleted and frozen (i.e., day 0 control for the screens). The library was left to expand 8 more days without doxycycline either in the presence of one of the 3 compounds (a total of 100 cells per sgRNA on average) or media only (250 cells per sgRNA). Cell concentration was assessed every 2 days and cells diluted back to 400 000 cells per ml whenever cell concentration was higher than 800 000 cells per ml. During this period, there were 7 to 8 population doublings for the untreated control while the cells treated with 1C8 had 3.7 doublings. All samples were then PBS-washed and cell pellets frozen. Genomic DNA was extracted using the QIAamp DNA blood maxi kit (Qiagen). sgRNA sequences were recovered and fitted with Illumina adaptors by PCR and NGS performed on an Illumina NextSeq 500 device (by the IRIC Genomics platform, see https://genomique.iric.ca) as previously described [58, 59]. Read counts of all DMSO and untreated control samples were summed to generate a single pooled control sgRNA distribution. Chemical-gene interaction scores were calculated by comparing the sgRNA read counts of the 1C8 screen to those of the control distribution using a version of RANKS [58] modified to control for the interaction between growth inhibition and the depletion of essential gene-targeting guides (Coulombe-Huntington et al., in preparation). This scoring approach ensures that fitness defects caused by gene loss independent of treatment do not confound the identification of bona fide chemical-genetic interactions. Genes showing no sgRNA depletion relative to the day 0 control and no enrichment relative to the day 8 control were assigned a score of zero.

### EMT and cell assays

MCF10a cells were maintained in 1:1 DMEM/F12 (Wisent), 20 ng/mL hEGF (Gibco), 0.5 μg/mL Hydrocortisone (Sigma-Aldrich), 10 μg/mL human recombinant insulin (Sigma-Aldrich), 5% Horse Serum (Gibco), and 1% Penicillin-Streptomycin (Wisent). MCF10a cells were plated onto 6 well plates at 3 × 10^5^ cells per well. Growth media was changed 24 hours after plating with media containing 4 ng/mL hTGFb-1 (Biolegend) or normal media, and 1C8 (10 μM final concentration), GPS167 (1 μM final concentration), or DMSO. Media was replenished 48 hours after treatment start. RNA was harvested at the time of treatment (D0) and 4 days later (D4) using Qiazol reagent (Qiagen) according to the manufacturer’s instructions. cDNA was generated using Superscript IV (Invitrogen) and qPCR was performed using PowerUp Sybr Green (Applied Biosystems). All expression values were normalized to the geometric mean of hTBP and hHPRT1 expression, and statistical tests performed with Tukey’s post-hoc test from triplicate experiments.

RT-qPCR primers used for EMT assay:

hsCDH2_F AGGCTTCTGGTGAAATCGCA

hsCDH2_R TGCAGTTGCTAAACTTCACATTG

hsCDH1_F CACCACGTACAAGGGTCAGG

hsCDH1_R GGTGTATACAGCCTCCCACG

hsVIM_F CGGGAGAAATTGCAGGAGGA

hsVIM_R AAGGTCAAGACGTGCCAGAG

hsTBP_F TGCACAGGAGCCAAGAGTGAA

hsTBP_R CACATCACAGCTCCCCACCA

hsHPRT1_F GAAAAGGACCCCACGAAGTGT

hsHPRT1_R AGTCAAGGGCATATCCTACAACA.

The CellTox Green cytotoxicity assay kit (Promega) was used according to instructions provided by the manufacturer. This assay is based on fluorescence signal enhancement upon binding of Green dye to DNA from compromised cells with impaired membrane integrity. Cells were monitored for cytotoxicity over 72 h after treatment on a fluorescence plate reader. DHX33 siRNA (CAAUGAAAGUCCCAAAUGUTT) oligos were transfected into HCT116 cells at a concentration of 80 nM using Lipofectamine 2000 (Invitrogen). Twenty-four hours after transfection, cells were treated with GPS167 and the CellTox Green cytotoxicity assay carried out.

## SUPPLEMENTARY MATERIALS







## Abbreviations

EMT: Epithelial Mesenchymal Transition
EKO: Extended Knockout
GSEA: Gene Set Enrichment Analysis
TGF-β: Transforming Growth Factor β
PSI: Percent Splicing Index
