# Supplementary material for: The anticancer potential of the CLK kinases inhibitors 1C8 and GPS167 revealed by their impact on the epithelial-mesenchymal transition and the antiviral immune response
Source: Oncotarget. 2024 May 16;15:313–25. doi: 10.18632/oncotarget.28585 (PMC11098031; doi:10.18632/oncotarget.28585)
Supplement: Supplementary file 1 [file oncotarget-15-28585-s001.pdf]

The anticancer potential of the CLK kinases inhibitors 1C8 and GPS167 revealed by their impact on the epithelial-mesenchymal transition and the antiviral immune response

SUPPLEMENTARY MATERIALS

Panel/Cell Line	Growth Percent
**Leukemia**	
CCRF-CEM	82.71
HL-60(TB)	54.93
K-562	78.32
MOLT-4	83.41
RPMI-8226	61.56
SR	55.92
**Non-Small Cell Lung Cancer**	
A549/ATCC	74.85
EKVX	81.73
HOP-62	96.37
HOP-92	41.18
NCI-H226	76.66
NCI-H23	91.3
NCI-H322M	45.15
NCI-H460	48.18
NCI-H522	53.28
**Colon Cancer**	
COLO 205	76.94
HCC-2998	86.99
HCT-116	56.23
HCT-15	54.88
HT29	68.21
KM12	65.32
SW-620	56.49
**CNS Cancer**	
SF-268	74.89
SF-295	109.91
SF-539	77.64
SNB-19	91.29
SNB-75	72.09
U251	63.15
**Melanoma**	
LOX IMVI	51.34
MALME-3M	65.65
M14	46.63
MDA-MB-435	75.61
SK-MEL-2	55.65
SK-MEL-28	78.25
SK-MEL-5	87.36
UACC-257	89.59
UACC-62	32.58
**Ovarian Cancer**	
IGROV1	27.34
OVCAR-3	75.55
OVCAR-4	53.4
OVCAR-5	83.96
OVCAR-8	59.25
NCI/ADR-RES	68.03
SK-OV-3	30.78
**Renal Cancer**	
786-0	56.24
A498	31.88
ACHN	32.58
CAKI-1	6.88
RXF 393	35.92
SN12C	75.31
TK-10	50.02
UO-31	28.12
**Prostate Cancer**	
PC-3	56.76
DU-145	63.23
**Breast Cancer**	
MCF7	34.7
MDA-MB-231/ATCC	47.9
HS 578T	54.72
BT-549	69.79
T-47D	74.61
MDA-MB-468	87.13
Mean	62.77
Delta	55.89
Range	103.03

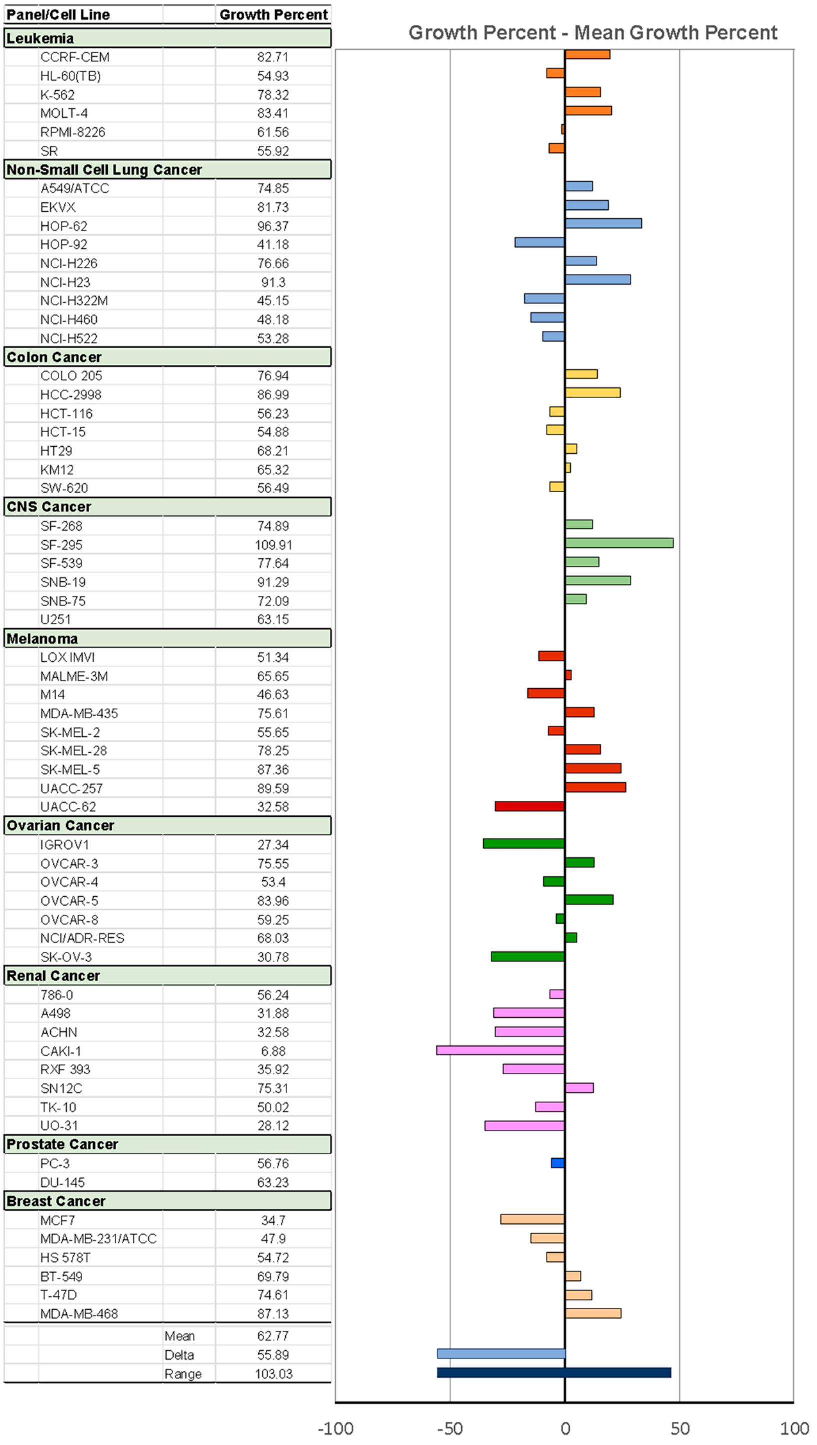

Supplementary Figure 1: Impact of 1C8 on various cancer cell lines. Ten µM of 1C8 was used to test the NCI-60 cancer cell lines panel. Values represent percentages of growth reduction or stimulation relative to DMSO-treated cells for the same period (48 hours).

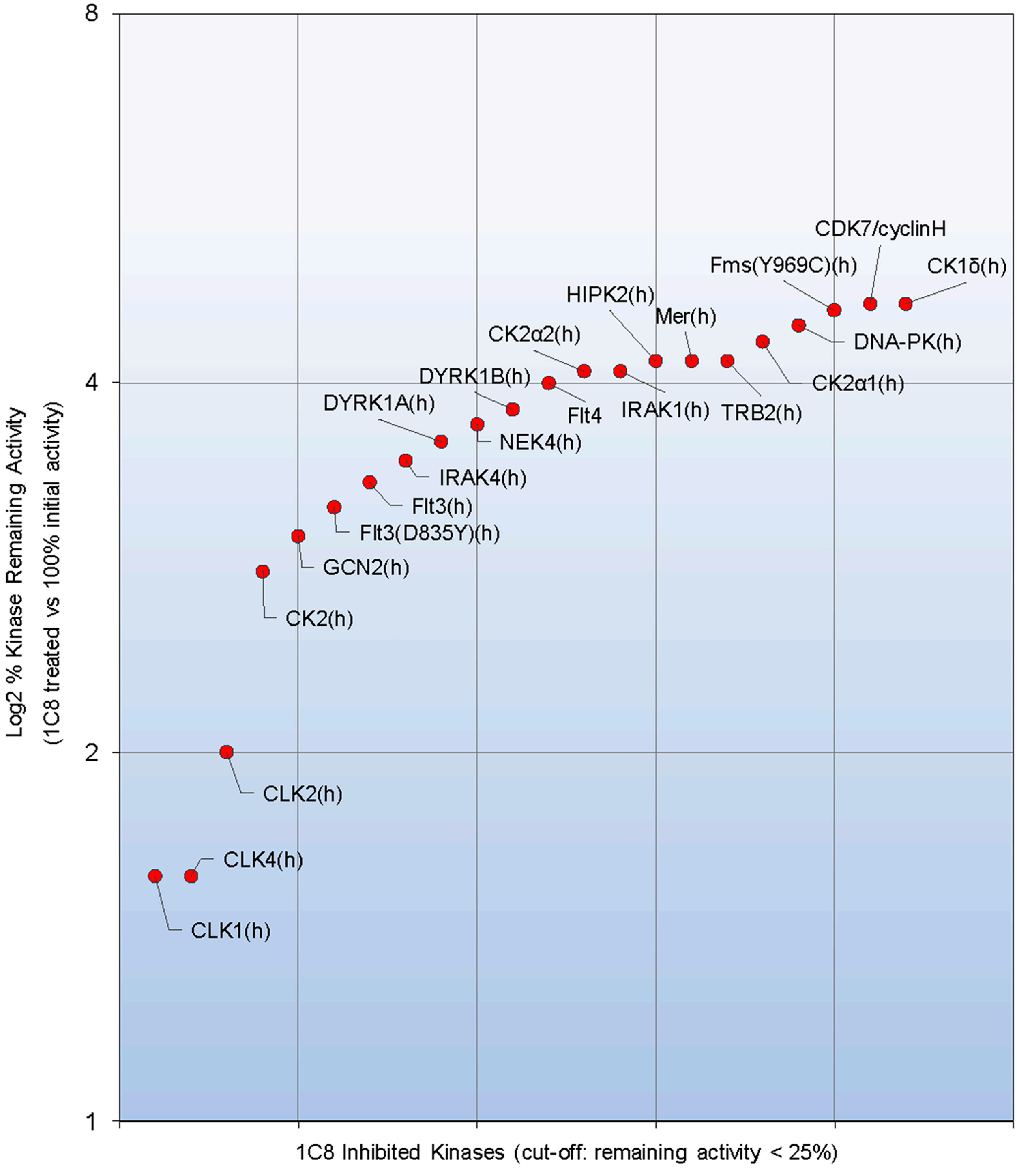

Supplementary Figure 2: Kinase inhibition by 1C8. The impact of 1C8 on kinase activity is reported only for kinases displaying less than 25% of remaining activity following incubation with 10 μM of 1C8. The assay was realized by Reaction Biology (United Kingdom).

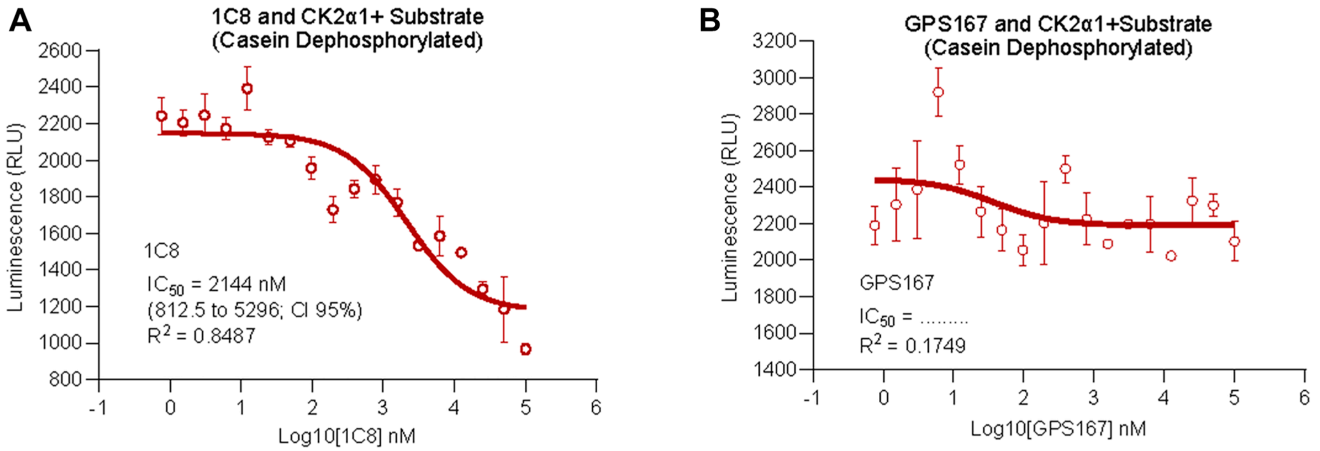

Supplementary Figure 3: Impact of 1C8 and GPS167 on CSNK2 (CK2) kinase activity. Impact of 1C8 (**A**) or GPS167 (**B**) on kinase activity of the recombinant full-length human active CK2α1 (SignalChem, C70-10G) in the presence of dephosphorylated casein (SignalChem C03-54BN) using the ADP-Glo™ Kinase Assay kit (Promega Cat # V6930). Determination of the IC50 inhibitory values for 1C8 and GPS167 was performed using the GraphPad Prism software (version 9.5.0).

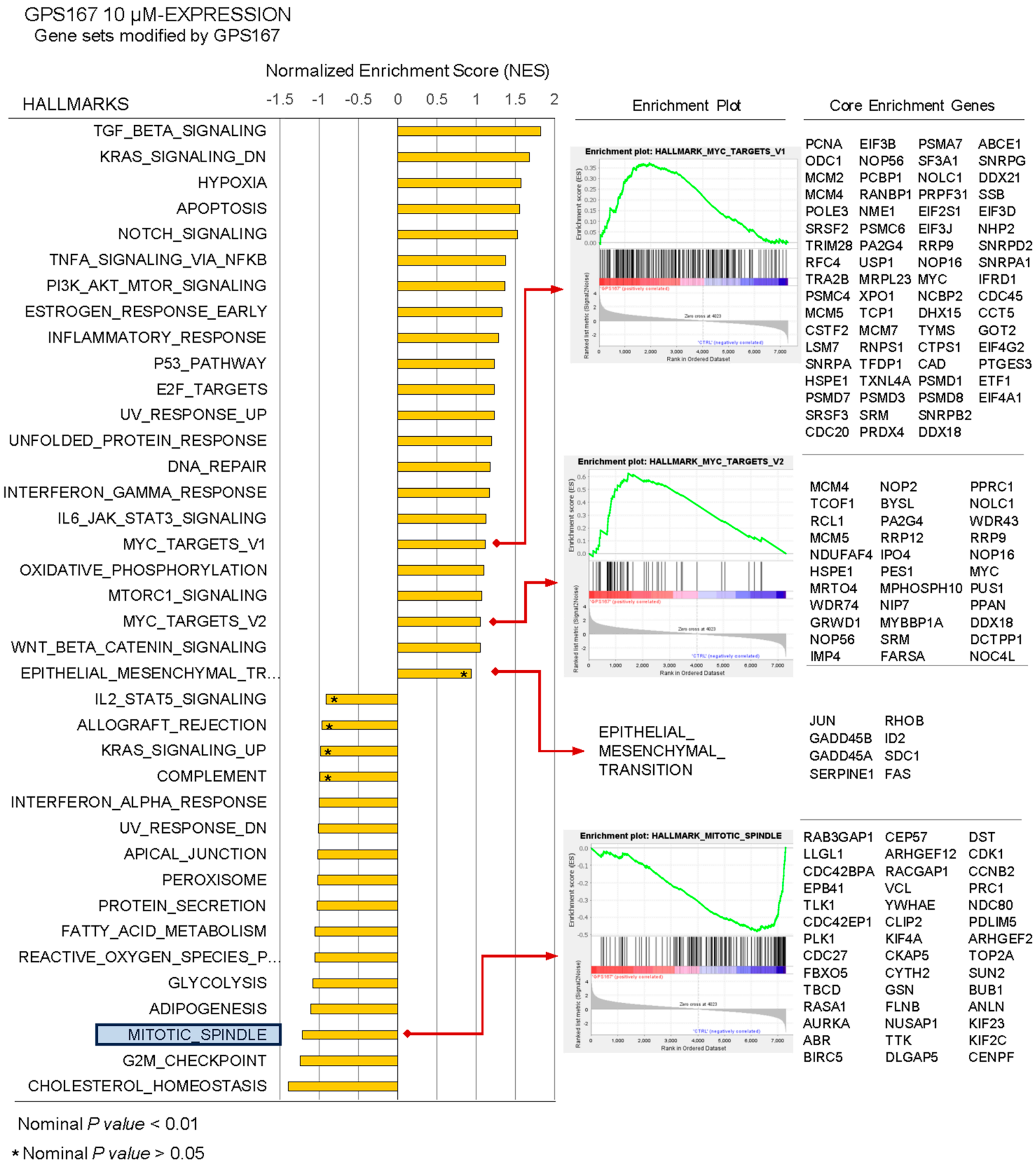

Supplementary Figure 4: Expression hits with GPS167. The expression analysis is based on a single sample of HCT116 cells treated with 10 µM of GPS167 collected after 24 hours and sequenced using Illumina protocols (Sohail et al., 2021**).** GSEA analysis using Hallmark gene sets from the Molecular Signature Database (MSigDB) was performed on genes differentially expressed relative to the controls. Normalized enrichment scores (NES) for categories of differentially expressed genes for 10 µM of GPS167 are shown with enrichment plots for 4 selected enriched gene sets with names of genes listed.

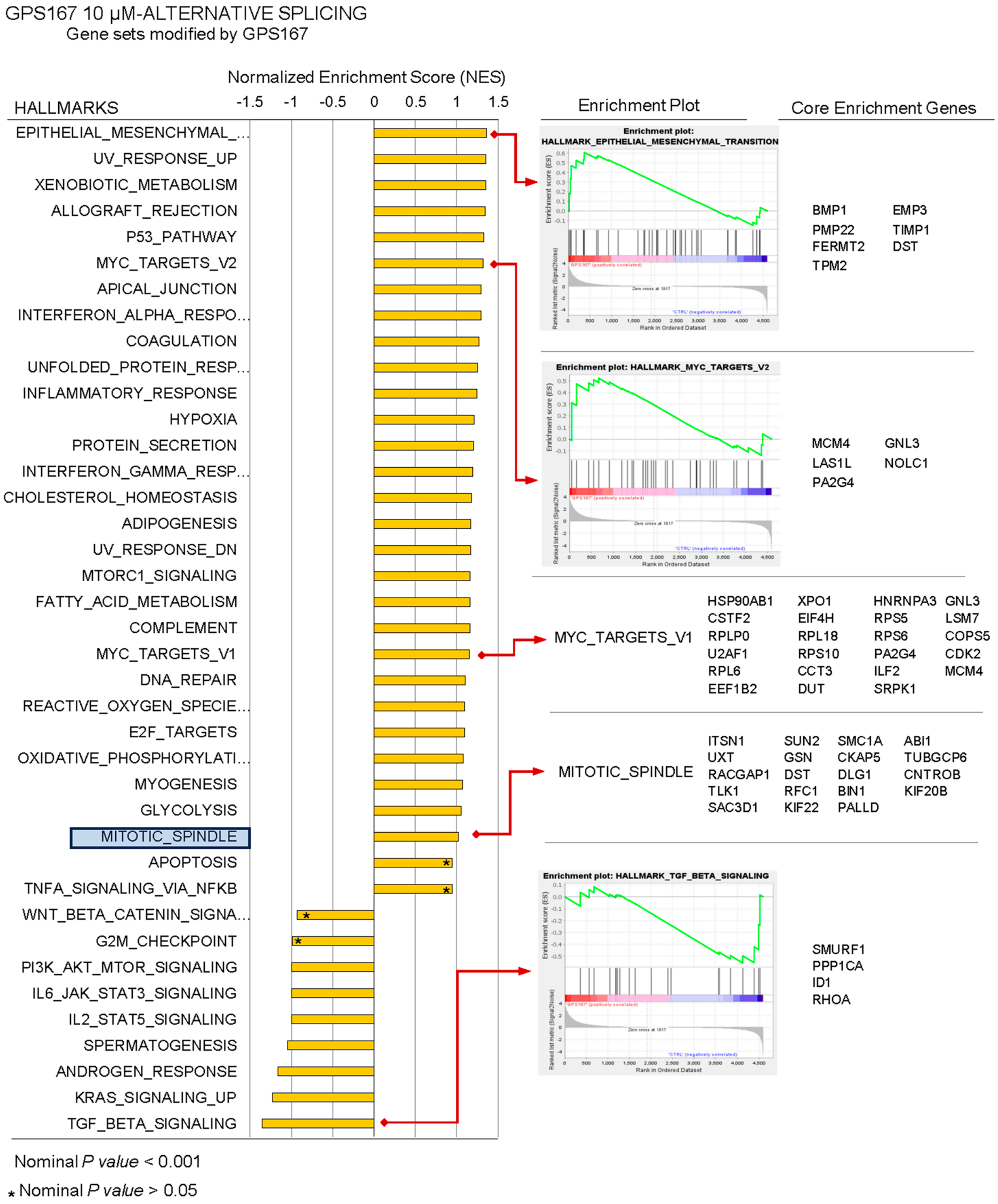

Supplementary Figure 5: Alternative splicing hits with GPS167. We used the RNAseq data performed on one sample of GPS167-treated HCT116 cells (Sohail et al. 2021) to identify alternative splicing events (ASEs) that changed relative to mock-treated cells. GSEA analysis was performed on differentially spliced transcripts relative to the controls. Hallmark gene sets from the Molecular Signature Database (MSigDB) were used for analysis. Normalized enrichment scores (NES) for categories of alternatively spliced genes for each dose of GPS167 are shown with enrichment plots for 6 selected gene sets. Names of genes in each set are listed.

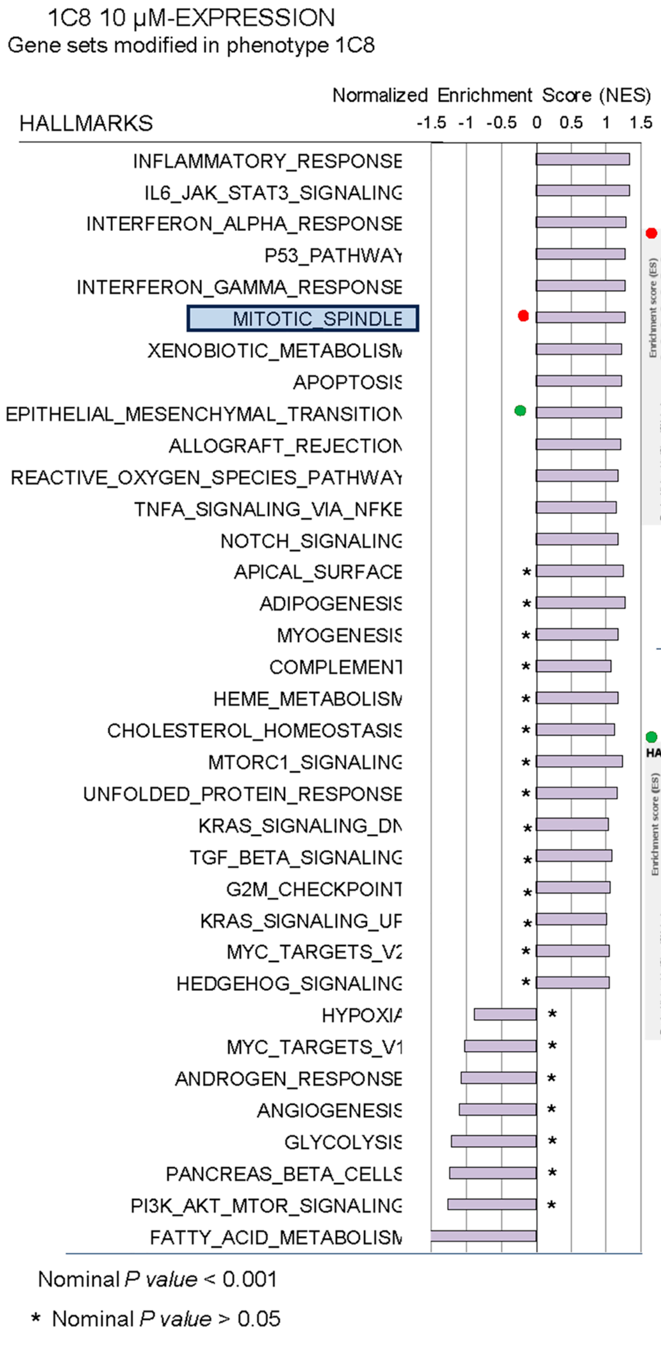

Enrichment Plot

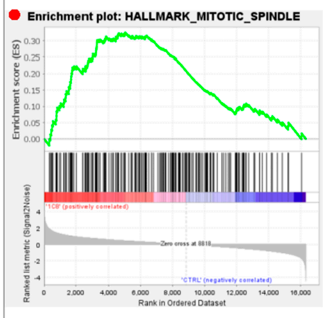

Core Enrichment Genes

DOCK2	DST	KIF4A	PRC1
TUBA4A	INCENP	RABGAP1	SHROOM1
ACTN4	MYH9	CCNB2	ARHGEF12
FLNA	BIN1	GEMIN4	EZR
MYO1E	MARCKS	STAU1	ARHGEF2
KIF3B	CDK5RAP2	CKAP5	ARF6
CLIP2	ARAP3	TSC1	SOS1
TPX2	CEP72	MARK4	PREX1
FSCN1	PALLD	ARL8A	CDC42EP4
BCAR1	CAPZB	APC	CYTH2
TRIO	CTTN	PLK1	MAP3K11
FARP1	CLASP1	SPTBN1	KIF2C
ARHGAP10	CLIP1	RALBP1	NEDD9
KIF3C	SAC3D1	AKAP13	AURKA
NOTCH2	VCL	CDC42BPA	KIF1B
ARHGAP29	DYNC1H1	PKD2	MYO9B
ABL1	LLGL1	PDLIM5	MID1
ARHGAP27	MID1IP1	DYNLL2	

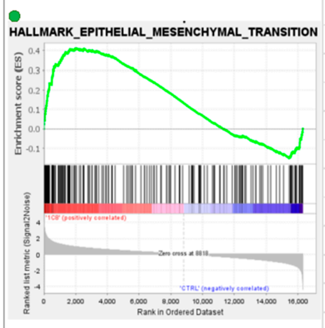

ACTA2	GREM1	GPC1	ABI3BP
ECM2	IL6	ADAM12	TAGLN
NNMT	MYLK	CCN2	LAMC2
THBS1	SDC1	FOXC2	CD44
MYL9	SCG2	FZD8	SERPINH1
TNFAIP3	WNT5A	FBLN5	CALD1
CDH6	PMP22	MMP2	MATN3
COL12A1	CCN1	TPM4	ITGB5
BASP1	FLNA	FAS	FSTL1
INHBA	TPM1	LAMA2	WIPF1
COL4A1	DAB2	COL6A2	CAPG
NOTCH2	DST	MEST	TPM2
CAP2	MAGEE1	FBLN1	VCAN

Supplementary Figure 6: Expression hits with 1C8. Triplicate samples of HeLa cells treated or not with 5, 10 and 20 µm of 1C8 were collected after 24 hours and sequenced using Illumina protocols ((Shkreta et al. 2017). A. Gene set enrichment analysis (GSEA) (Mootha et al. 2003; Subramanian et al. 2005) was performed on genes differentially expressed relative to the controls. Hallmark gene sets from the Molecular Signature Database (MSigDB) were used for analysis. Normalized enrichment scores (NES) for categories of differentially expressed genes are shown with enrichment plots for the mitotic spindle and EMT sets. Names of genes in each set are listed.

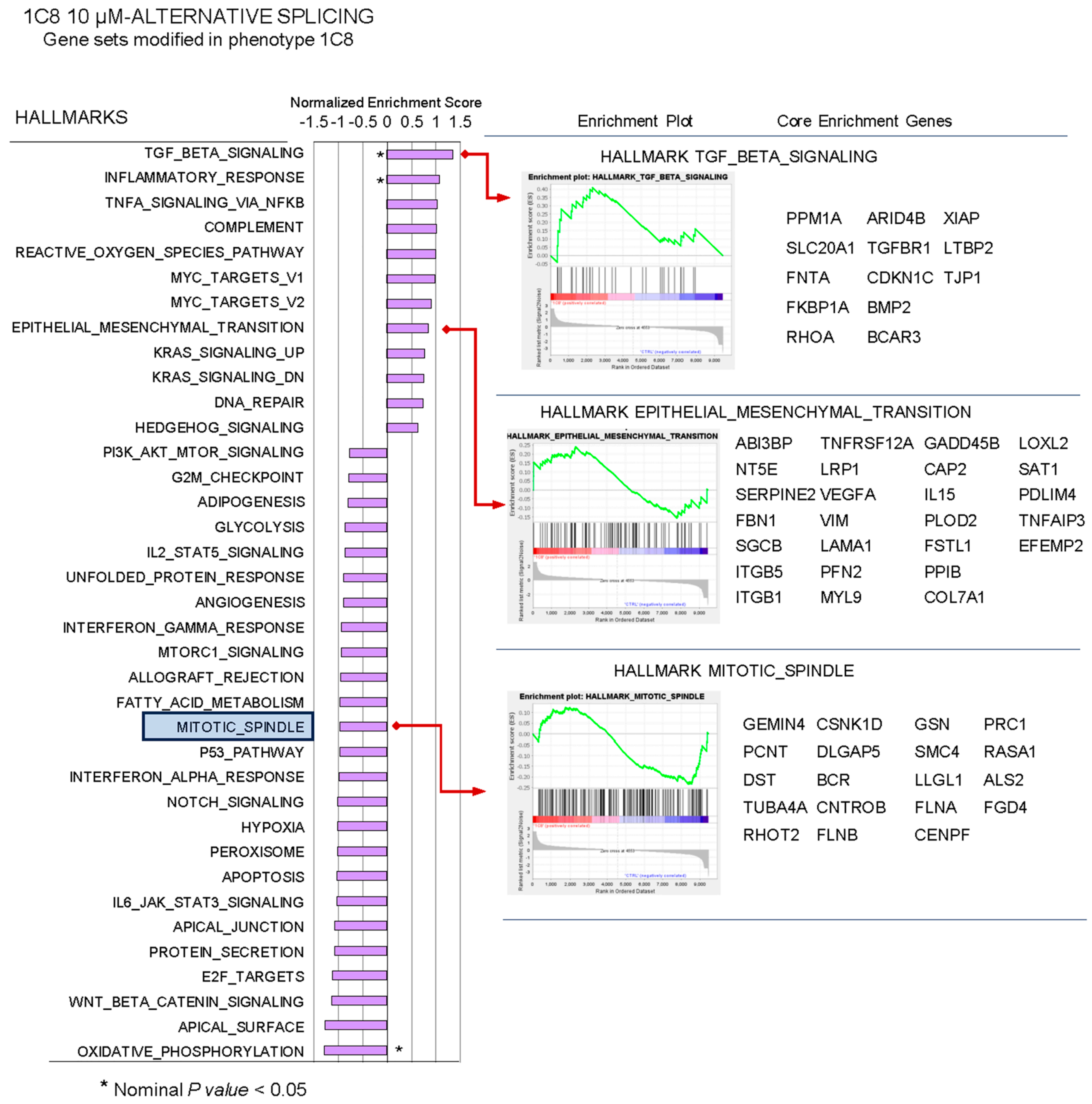

Supplementary Figure 7: Alternative splicing hits with 1C8. Samples were analyzed for alternative splicing based on inclusion values of specific alternative splicing events (ASEs). PSI refers to per-splicing index which measures the relative amount of the long variant relative to the sum of both short and long. GSEA was performed on differentially spliced transcripts relative to the controls. Hallmark gene sets from the Molecular Signature Database (MSigDB) were used for analysis. Normalized enrichment scores (NES) for categories of alternatively spliced genes are shown. Enrichment plots for three of the most significantly enriched gene sets. Names of genes in each set are listed.

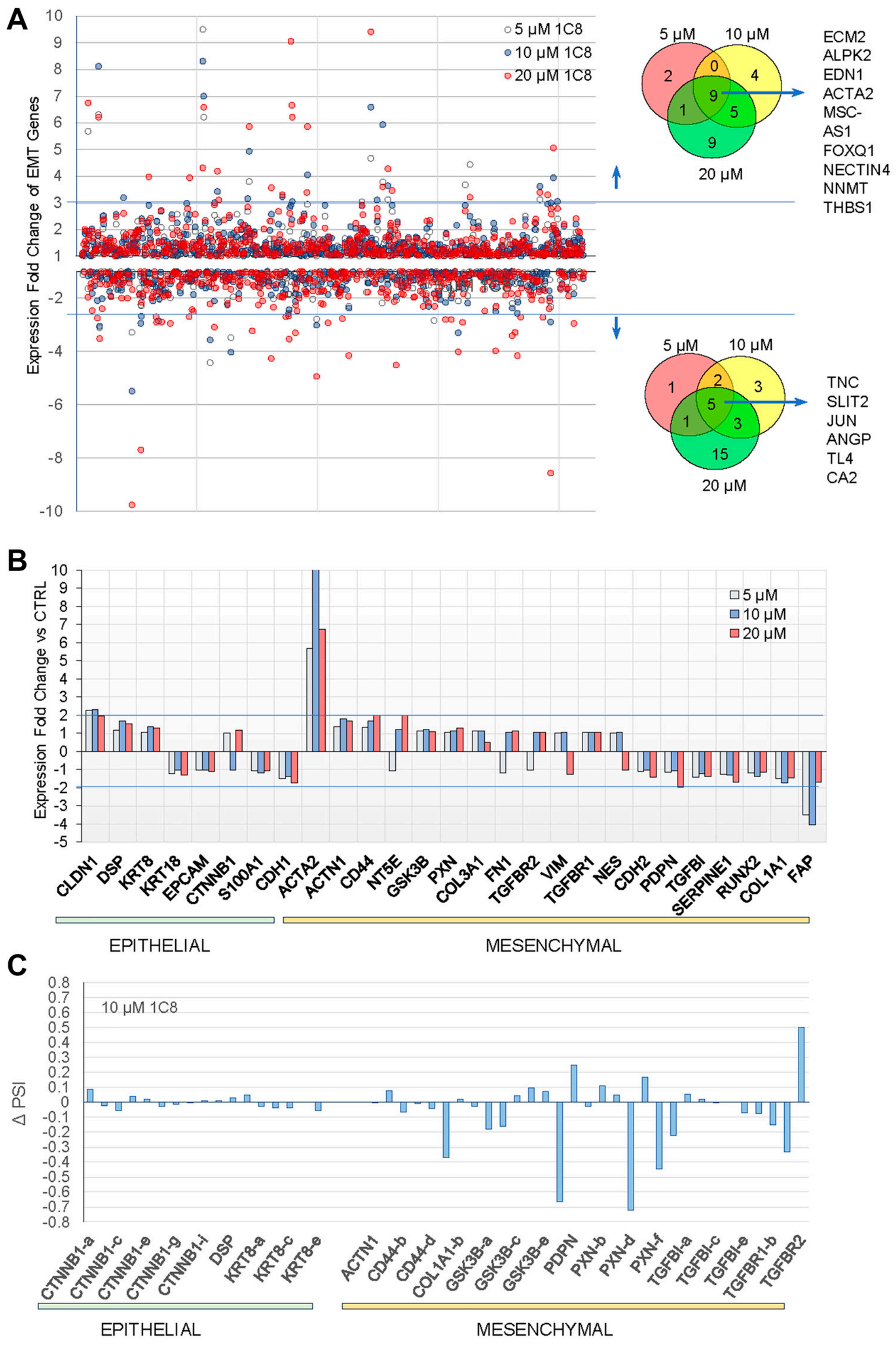

Supplementary Figure 8: 1C8 and EMT. (**A**) Impact of 5 µM, 10 µM and 20 µM of 1C8 on the expression in HCT116 cells of EMT signature genes from the EMTome platform (http://www.emtome.org/). The Venn diagrams show top genes whose expression changed >3-fold. (**B**) Impact of 1C8 on the expression of genes categorized as epithelial or mesenchymal markers according to various published sources. (**C**) Change in the alternative splicing of epithelial and mesenchymal marker genes following treatment of HCT116 cells with 10 µM 1C8.

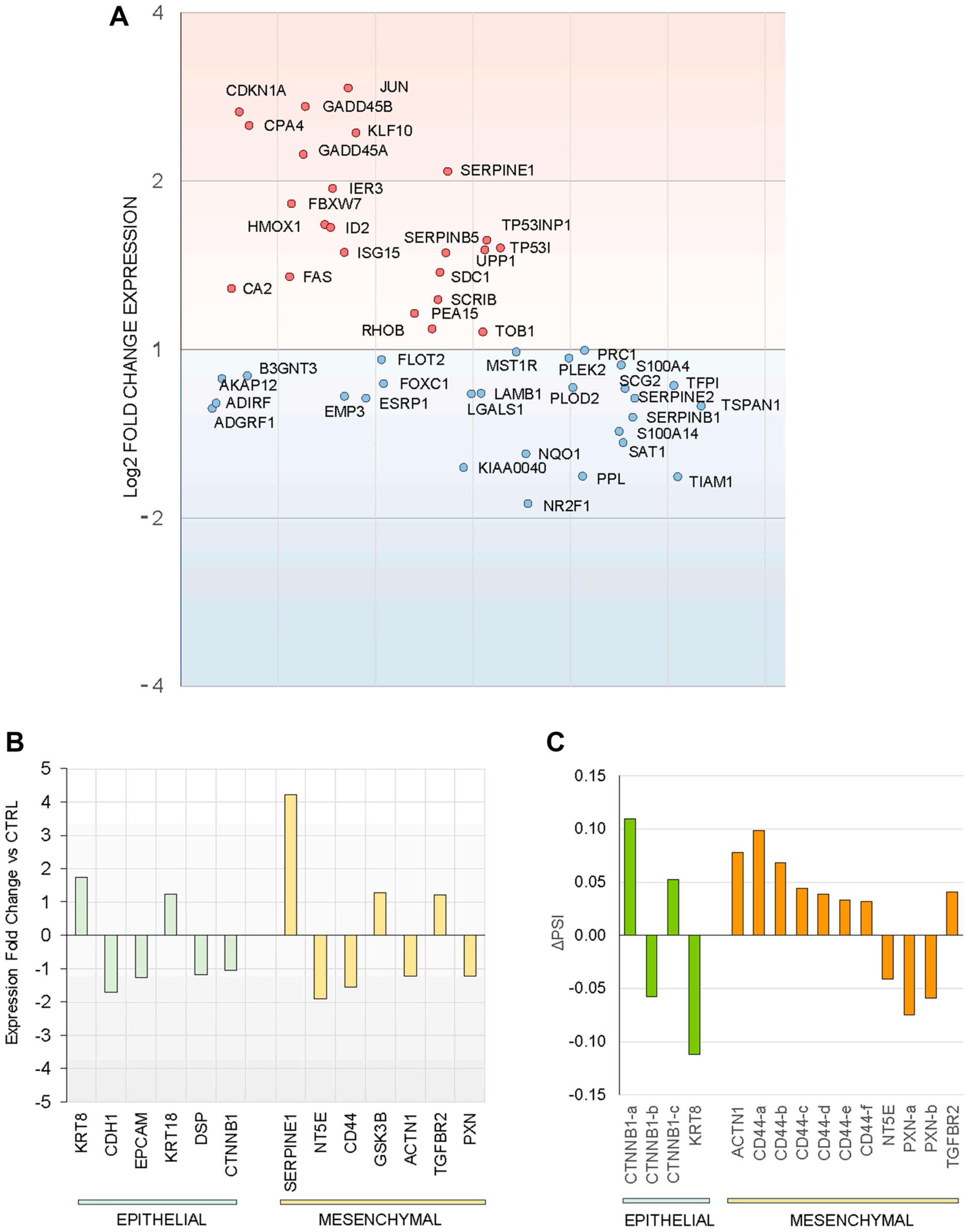

Supplementary Figure 9: GPS167 and EMT. (**A**) Impact of 10 µM GPS167 on the expression of EMT signature genes (EMTome). Only genes whose expression changed by more than 2-fold are shown. (**B**) GPS167 induced changes on the expression of epithelial and mesenchymal marker genes. (**C**) Impact of treating HCT116 cells with 10 µM GPS167 on the alternative splicing of selected epithelial and mesenchymal marker genes.

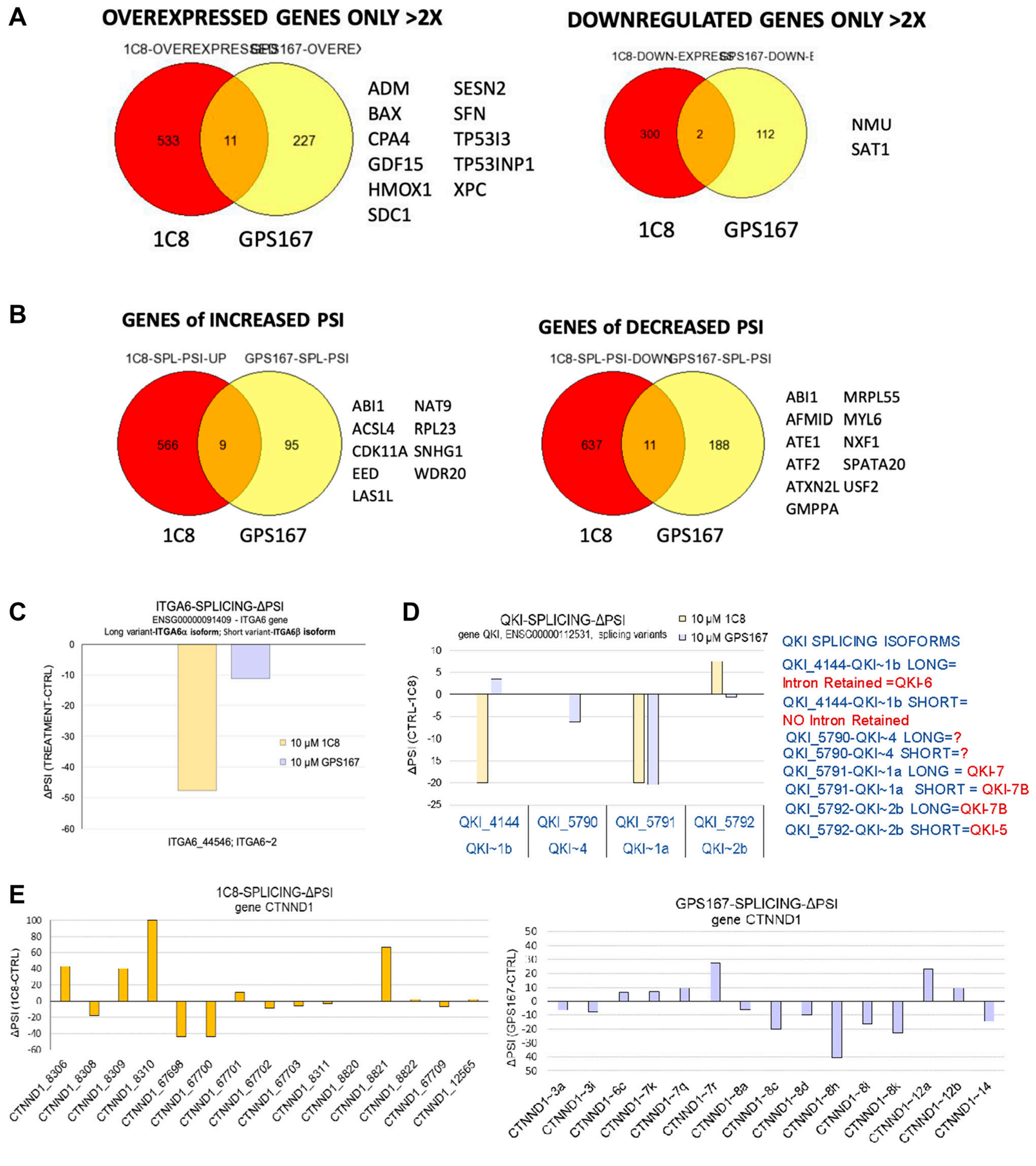

Supplementary Figure 10: Common hits of GPS167 and 1C8. (**A**) The Venn diagrams show the number of genes in HCT116 cells whose expression was increased (left) or decreased (right) more than two-fold by treatment with 1C8 or GPS167 drugs at 10 µM. The number and names of genes whose expression was affected by both drugs are indicated. (**B**) Common alternative splicing hits of 1C8 and GPS167. The Venn diagram of the left indicates the number of genes whose PSI significantly ($p < 0.05$) increased or decreased (right panel) with 1C8 or GPS167 as compared to DMSO-treated HCT116 cells. The number and the names of genes whose alternative splicing is affected by both 1C8 and GPS167 are indicated. Impact of 1C8 and GPS167 on the alternative splicing of *ITGA6* (**C**), *QKI* (**D**) and *CTNND1* (**E**).

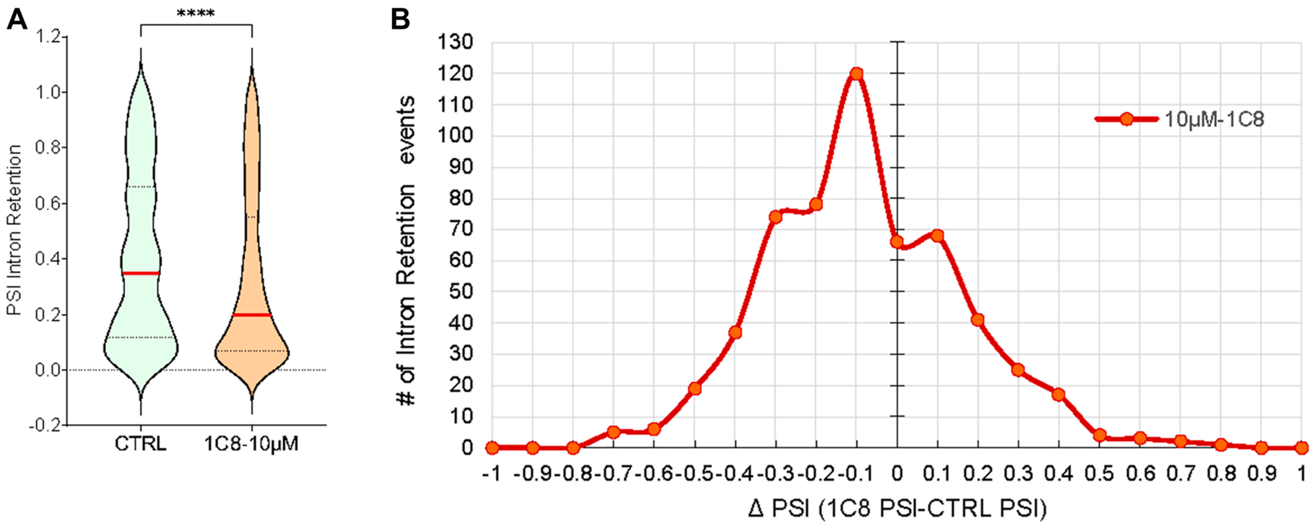

Supplementary Figure 11: Intron-retention events affected by 1C8 and GPS167. (**A**) Violin plots depict the distribution of PSIs of intron retention (IR) events in untreated and 1C8-treated HCT116 cells. Only PSI values for the same intron retention event of *p*-value < 0.05 determined for triplicates of control and 1C8-treated HCT116 cells are plotted. The mean values are shown by a red line while quartiles are indicated by an interrupted black line. Two-way ANOVA analysis is performed using GraphPad Prism software ($^{****}p < 0.0001$). (**B**) Distribution of ΔPSI frequency of IR events ($p < 0.05$) following 1C8 treatment of HCT116 cells.

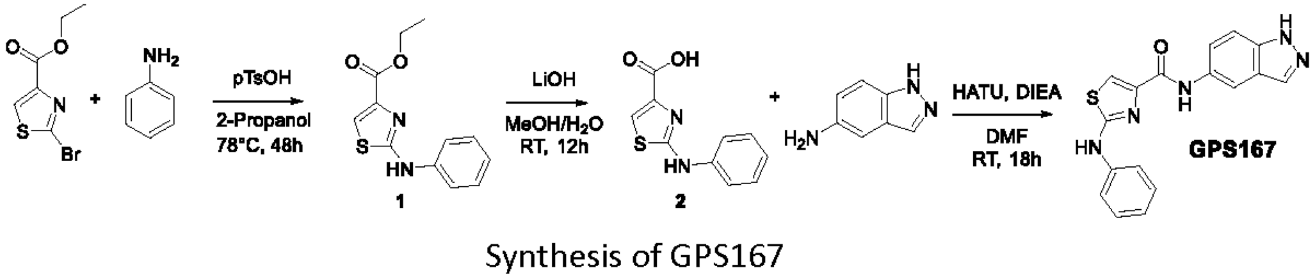

Supplementary Figure 12: Synthesis of GPS167.

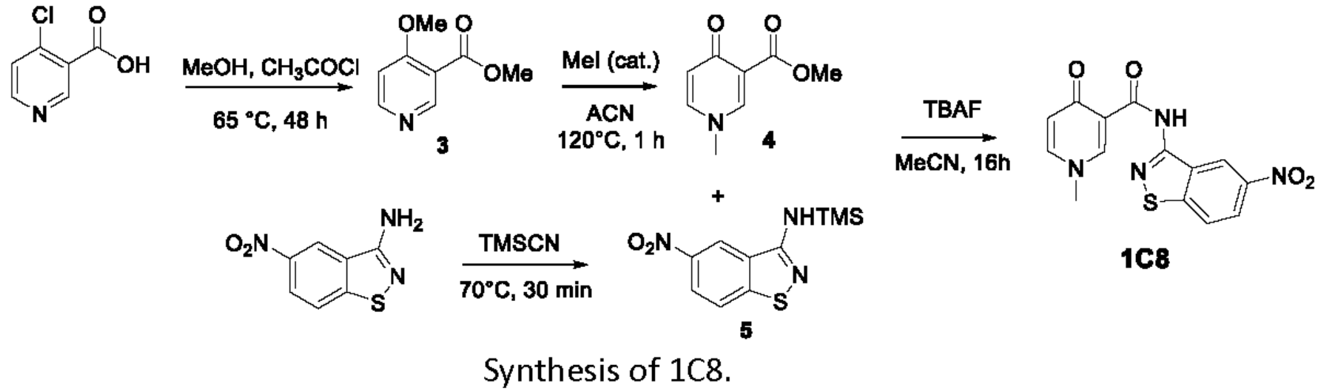

Supplementary Figure 13: Synthesis of 1C8.

Supplementary Table 1: Inhibition of kinases by 1C8. See Supplementary Table 1

Supplementary Table 2: Gene list for the chemogenomic screen. See Supplementary Table 2
