# Supplementary material for: The anticancer potential of the CLK kinases inhibitors 1C8 and GPS167 revealed by their impact on the epithelial-mesenchymal transition and the antiviral immune response
Source: Oncotarget. 2024 May 16;15:313–25. doi: 10.18632/oncotarget.28585 (PMC11098031; doi:10.18632/oncotarget.28585)
Supplement: Supplementary file 2 [file oncotarget-15-28585-s002.docx]

**Supplementary Table 1: Inhibition of kinases by 1C8**

| **KINASES** | **1C8 @ 10 µM** | **KINASES** | **1C8 @ 10 µM** | **KINASES** | **1C8 @ 10 µM** |
| --- | --- | --- | --- | --- | --- |
| AAK1(h) | 49 | Flt3(h) | 10 | PKBγ(h) | 88 |
| Abl(h) | 97 | Flt4(h) | 16 | PKCα(h) | 91 |
| Abl(m) | 83 | Fms(h) | 35 | PKCβI(h) | 95 |
| Abl (H396P) (h) | 79 | Fms(Y969C)(h) | 24 | PKCβII(h) | 88 |
| Abl (M351T)(h) | 76 | Fyn(h) | 66 | PKCγ(h) | 71 |
| Abl (Q252H) (h) | 78 | GCK(h) | 70 | PKCδ(h) | 100 |
| Abl(T315I)(h) | 99 | GCN2(h) | 8 | PKCε(h) | 97 |
| Abl(Y253F)(h) | 67 | GRK1(h) | 97 | PKCη(h) | 99 |
| ACK1(h) | 80 | GRK2(h) | 105 | PKCι(h) | 73 |
| ACTR2(h) | 105 | GRK3(h) | 92 | PKCμ(h) | 102 |
| ALK(h) | 82 | GRK5(h) | 117 | PKCθ(h) | 98 |
| ALK1(h) | 99 | GRK6(h) | 92 | PKCζ(h) | 96 |
| ALK2(h) | 126 | GRK7(h) | 101 | PKD2(h) | 97 |
| ALK4(h) | 101 | GSK3α(h) | 58 | PKD3(h) | 126 |
| ALK6(h) | 111 | GSK3β(h) | 77 | PKG1α(h) | 106 |
| Arg(h) | 84 | Haspin(h) | 80 | PKG1β(h) | 102 |
| AMPKα1(h) | 92 | Hck(h) | 90 | PKR(h) | 34 |
| AMPKα2(h) | 90 | Hck(h) activated | 74 | Plk1(h) | 81 |
| A-Raf(h) | 74 | HIPK1(h) | 37 | Plk3(h) | 124 |
| Arg(m) | 77 | HIPK2(h) | 18 | Plk4(h) | 85 |
| ARK5(h) | 85 | HIPK3(h) | 45 | PRAK(h) | 89 |
| ASK1(h) | 92 | HIPK4(h) | 40 | PRKG2(h) | 83 |
| Aurora-A(h) | 69 | HPK1(h) | 95 | PRK1(h) | 106 |
| Aurora-B(h) | 78 | HRI(h) | 100 | PRK2(h) | 99 |
| Aurora-C(h) | 84 | ICK(h) | 54 | PrKX(h) | 91 |
| Axl(h) | 45 | IGF-1R(h) | 107 | PRP4(h) | 93 |
| BIKe(h) | 53 | IGF-1R(h), activated | 88 | PTK5(h) | 65 |
| Blk(h) | 85 | IKKα(h) | 89 | Pyk2(h) | 83 |
| Blk(m) | 86 | IKKβ(h) | 82 | Ret(h) | 98 |
| BMPR2(h) | 99 | IKKε(h) | 93 | Ret (V804L)(h) | 69 |
| Bmx(h) | 82 | IR(h) | 97 | Ret(V804M)(h) | 66 |
| BRK(h) | 97 | IR(h), activated | 70 | RIPK1(h) | 107 |
| BrSK1(h) | 91 | IRE1(h) | 89 | RIPK2(h) | 77 |
| BrSK2(h) | 96 | IRR(h) | 84 | ROCK-I(h) | 100 |
| BTK(h) | 81 | IRAK1(h) | 17 | ROCK-II(h) | 93 |
| BTK(R28H)(h) | 92 | IRAK4(h) | 11 | ROCK-II(r) | 93 |
| B-Raf(h) | 93 | Itk(h) | 65 | Ron(h) | 120 |
| B-Raf(V599E)(h) | 92 | JAK1(h) | 80 | Ros(h) | 113 |
| CaMKI(h) | 96 | JAK2(h) | 105 | Rse(h) | 92 |
| CaMKIß(h) | 108 | JAK3(h) | 93 | Rsk1(h) | 77 |
| CaMKIγ(h) | 95 | JNK1α1(h) | 87 | Rsk1(r) | 80 |
| CaMKIIα(h) | 52 | JNK2α2(h) | 72 | Rsk2(h) | 84 |
| CaMKIIβ(h) | 84 | JNK3(h) | 34 | Rsk3(h) | 88 |
| CaMKIIγ(h) | 57 | KDR(h) | 42 | Rsk4(h) | 76 |
| CaMKIδ(h) | 105 | Lck(h) | 91 | SAPK2a(h) | 97 |
| CaMKIIδ(h) | 91 | Lck(h) activated | 63 | SAPK2a(T106M)(h) | 97 |
| CaMKIV(h) | 79 | LIMK1(h) | 88 | SAPK2b(h) | 98 |
| CaMKK1(h) | 76 | LIMK2(h) | 86 | SAPK3(h) | 115 |
| CaMKK2(h) | 70 | LKB1(h) | 78 | SAPK4(h) | 93 |
| Cdc7/cyclinB1(h) | 75 | LOK(h) | 86 | SBK1(h) | 116 |
| CDK1/cyclinB(h) | 77 | Lyn(h) | 91 | SGK(h) | 90 |
| CDK2/cyclinA(h) | 84 | Lyn(m) | 74 | SGK2(h) | 98 |
| CDK2/cyclinE(h) | 87 | LRRK2(h) | 95 | SGK3(h) | 106 |
| CDK3/cyclinE(h) | 90 | LTK(h) | 77 | SIK(h) | 85 |
| CDK4/cyclinD3(h) | 102 | MAK(h) | 74 | SIK2(h) | 112 |
| CDK5/p25(h) | 84 | MAPK1(h) | 96 | SIK3(h) | 95 |
| CDK5/p35(h) | 71 | MAPK2(h) | 99 | SLK(h) | 87 |
| CDK6/cyclinD3(h) | 91 | MAPK2(m) | 96 | Snk(h) | 94 |
| CDK7/cyclinH/MAT1(h) | 25 | MAP4K3(h) | 102 | SNRK(h) | 103 |
| CDK9/cyclin T1(h) | 33 | MAP4K4(h) | 72 | Src(1-530)(h) | 78 |
| CDK12/cyclinK(h) | 82 | MAP4K5(h) | 92 | Src(T341M)(h) | 104 |
| CDK13/cyclinK(h) | 85 | MAPKAP-K2(h) | 100 | SRMS(h) | 89 |
| CDK14/cyclinY(h) | 54 | MAPKAP-K3(h) | 91 | SRPK1(h) | 64 |
| CDK16/cyclinY(h) | 50 | MEK1(h) | 105 | SRPK2(h) | 84 |
| CDK17/cyclinY(h) | 74 | MEK2(h) | 98 | STK16(h) | 33 |
| CDK18/cyclinY(h) | 35 | MARK1(h) | 95 | STK25(h) | 86 |
| CDKL1(h) | 90 | MARK3(h) | 95 | STK32A(h) | 95 |
| CDKL2(h) | 74 | MARK4(h) | 83 | STK32B(h) | 104 |
| CDKL3(h) | 34 | MEKK2(h) | 105 | STK32C(h) | 103 |
| CDKL4(h) | 34 | MEKK3(h) | 99 | STK33(h) | 56 |
| ChaK1(h) | 106 | MELK(h) | 42 | Syk(h) | 98 |
| CHK1(h) | 99 | Mer(h) | 18 | TAF1L(h) | 36 |
| CHK2(h) | 37 | Met(h) | 67 | TAK1(h) | 99 |
| CHK2(I157T)(h) | 42 | Met(D1246H)(h) | 63 | TAO1(h) | 102 |
| CHK2(R145W)(h) | 50 | Met(D1246N)(h) | 58 | TAO2(h) | 88 |
| CK1ε(h) | 31 | Met(M1268T)(h) | 93 | TAO3(h) | 103 |
| CK1γ1(h) | 69 | Met(Y1248C)(h) | 87 | TBK1(h) | 108 |
| CK1γ2(h) | 50 | Met(Y1248D)(h) | 108 | Tec(h) activated | 84 |
| CK1γ3(h) | 41 | Met(Y1248H)(h) | 100 | TGFBR1(h) | 98 |
| CK1δ(h) | 25 | MINK(h) | 97 | TGFBR2(h) | 68 |
| CK1(y) | 87 | MKK3(h) | 104 | Tie2 (h) | 93 |
| CK2(h) | 7 | MKK4(m) | 85 | Tie2(R849W)(h) | 94 |
| CK2α1(h) | 20 | MKK6(h) | 98 | Tie2(Y897S)(h) | 99 |
| CK2α2(h) | 17 | MLCK(h) | 89 | TLK1(h) | 106 |
| CLIK1(h) | 77 | MLK1(h) | 58 | TLK2(h) | 88 |
| CLK1(h) | 3 | MLK2(h) | 106 | TNIK(h) | 92 |
| CLK2(h) | 4 | MLK3(h) | 94 | TRB2(h) | 18 |
| CLK3(h) | 59 | Mnk2(h) | 82 | TrkA(h) | 41 |
| CLK4(h) | 3 | MOK(h) | 86 | TrkB(h) | 75 |
| cKit(h) | 76 | MRCKα(h) | 96 | TrkC(h) | 110 |
| cKit(D816V)(h) | 70 | MRCKβ(h) | 91 | TSSK1(h) | 78 |
| cKit(D816H)(h) | 83 | MRCKγ(h) | 93 | TSSK2(h) | 94 |
| cKit(V560G)(h) | 28 | MSK1(h) | 88 | TSSK3(h) | 121 |
| cKit(V654A)(h) | 30 | MSK2(h) | 92 | TSSK4(h) | 94 |
| CRIK(h) | 117 | MSSK1(h) | 112 | TTBK1(h) | 94 |
| CSK(h) | 100 | MST1(h) | 97 | TTBK2(h) | 100 |
| c-RAF(h) | 92 | MST2(h) | 100 | TTK(h) | 94 |
| cSRC(h) | 71 | MST3(h) | 98 | Txk(h) | 59 |
| DAPK1(h) | 75 | MST4(h) | 96 | TYK2(h) | 92 |
| DAPK2(h) | 84 | mTOR(h) | 95 | ULK1(h) | 90 |
| DCAMKL2(h) | 68 | mTOR/FKBP12(h) | 91 | ULK2(h) | 98 |
| DCAMKL3(h) | 114 | MuSK(h) | 94 | ULK3(h) | 91 |
| DDR1(h) | 69 | MYLK2(h) | 76 | VRK1(h) | 102 |
| DDR2(h) | 105 | MYO3B(h) | 87 | VRK2(h) | 101 |
| DMPK(h) | 91 | NDR2(h) | 84 | Wee1(h) | 110 |
| DRAK1(h) | 79 | NEK1(h) | 87 | Wee1B(h) | 90 |
| DRAK2(h) | 94 | NEK2(h) | 88 | WNK1(h) | 114 |
| DYRK1A(h) | 12 | NEK4(h) | 13 | WNK2(h) | 86 |
| DYRK1B(h) | 14 | NEK3(h) | 96 | WNK3(h) | 100 |
| DYRK2(h) | 82 | NEK6(h) | 99 | WNK4(h) | 102 |
| DYRK3(h) | 51 | NEK7(h) | 85 | Yes(h) | 75 |
| eEF-2K(h) | 109 | NEK9(h) | 96 | ZAK(h) | 84 |
| EGFR(h) | 117 | NIM1(h) | 89 | ZAP-70(h) | 121 |
| EGFR(L858R)(h) | 105 | NEK11(h) | 47 | ZIPK(h) | 82 |
| EGFR(L861Q)(h) | 84 | NLK(h) | 91 | ATM(h) | 91 |
| EGFR(T790M)(h) | 94 | NUAK2(h) | 86 | ATR/ATRIP(h) | 98 |
| EGFR(T790M,L858R)(h) | 52 | p70S6K(h) | 74 | DNA-PK(h) | 22 |
| EphA1(h) | 99 | PAK1(h) | 94 | PI3 Kinase (p110b/p85a)(h) | 71 |
| EphA2(h) | 108 | PAK2(h) | 89 | PI3 Kinase (p120g)(h) | 91 |
| EphA3(h) | 86 | PAK4(h) | 96 | PI3 Kinase (p110d/p85a)(h) | 48 |
| EphA4(h) | 83 | PAK3(h) | 97 | PI3 Kinase (p110a/p85a)(m) | 77 |
| EphA5(h) | 77 | PAK5(h) | 92 | PI3 Kinase (p110a/p65a)(m) | 82 |
| EphA7(h) | 91 | PAK6(h) | 124 | PI3 Kinase (p110a(E545K)/p85a)(m) | 76 |
| EphA8(h) | 101 | PAR-1Bα(h) | 84 | PI3 Kinase (p110a(H1047R)/p85a)(m) | 58 |
| EphB2(h) | 77 | PASK(h) | 68 | PI3 Kinase (p110b/p85b)(m) | 95 |
| EphB1(h) | 122 | PEK(h) | 75 | PI3 Kinase (p110b/p85a)(m) | 66 |
| EphB3(h) | 92 | PDGFRα(h) | 94 | PI3 Kinase (p110d/p85a)(m) | 94 |
| EphB4(h) | 98 | PDGFRα(D842V)(h) | 41 | PI3 Kinase (p110a(E542K)/p85a)(m) | 88 |
| ErbB2(h) | 85 | PDGFRα(V561D)(h) | 81 | PI3 Kinase (p110a/p85a)(h) | 70 |
| ErbB4(h) | 101 | PDGFRβ(h) | 85 | PI3 Kinase (p110a(E542K)/p85a)(h) | 80 |
| FAK(h) | 84 | PDHK2(h) | 99 | PI3 Kinase (p110a(H1047R)/p85a)(h) | 79 |
| Fer(h) | 91 | PDHK4(h) | 88 | PI3 Kinase (p110a(E545K)/p85a)(h) | 86 |
| Fes(h) | 65 | PDK1(h) | 110 | PI3 Kinase (p110a/p65a)(h) | 82 |
| FGFR1(h) | 111 | PhKγ1(h) | 51 | PI3KC2a(h) | 87 |
| FGFR1(V561M)(h) | 98 | PhKγ2(h) | 67 | PI3KC2g(h) | 55 |
| FGFR2(h) | 94 | Pim-1(h) | 43 | PIP4K2a(h) | 91 |
| FGFR2(N549H)(h) | 90 | Pim-2(h) | 39 | PIP5K1a(h) | 98 |
| FGFR3(h) | 88 | Pim-3(h) | 42 | PIP5K1g(h) | 97 |
| FGFR4(h) | 94 | PKA(h) | 86 |  |  |
| Fgr(h) | 63 | PKAcβ(h) | 116 |  |  |
| Flt1(h) | 80 | PKBα(h) | 95 |  |  |
| Flt3(D835Y)(h) | 9 | PKBβ(h) | 93 |  |  |

The impact of 1C8 on the activity of 421 kinases is reported as percentage of initial activity following incubation with 10 µM of 1C8. The assay was realized by Reaction Biology (United Kingdom).
