# Supplementary material for: The anticancer potential of the CLK kinases inhibitors 1C8 and GPS167 revealed by their impact on the epithelial-mesenchymal transition and the antiviral immune response
Source: Oncotarget. 2024 May 16;15:313–25. doi: 10.18632/oncotarget.28585 (PMC11098031; doi:10.18632/oncotarget.28585)
Supplement: Supplementary file 3 [file oncotarget-15-28585-s003.docx]

| **Supplementary Table 2: Gene list for the chemogenomic screen** | | | | | | | |  |
| --- | --- | --- | --- | --- | --- | --- | --- | --- |
| **Gene** | **RANKS_score** | **p-value** | **Gene** | **RANKS_score** | **p-value** | **Gene** | **RANKS_score** | **p-value** |
| GCN1L1 | 3.41 | 1.00E-06 | PCDH15 | 1.42 | 0.013997 | WTH3DI | 1.23 | 0.031874 |
| MBTPS2 | 3.5 | 1.00E-06 | ANKRD52 | 1.42 | 0.013997 | GNPAT | 1.23 | 0.031874 |
| EIF2AK4 | 3.61 | 1.00E-06 | CHCHD1 | 1.42 | 0.013997 | CLEC16A | 1.23 | 0.031874 |
| TCF4 | -3.33 | 1.00E-06 | PTGES3 | 1.42 | 0.013997 | RAVER2 | 1.23 | 0.031874 |
| NSD1 | 3.02 | 5.00E-06 | MYL6B | 1.42 | 0.013997 | FXR1 | 1.23 | 0.031874 |
| SCAP | 2.82 | 6.00E-06 | DHRSX | 1.42 | 0.013997 | CWC27 | -1.23 | 0.031874 |
| MAD2L2 | -2.91 | 1.00E-05 | MRPL39 | 1.42 | 0.013997 | UBA7 | -1.23 | 0.031874 |
| WARS2 | 2.61 | 2.20E-05 | IDH1 | 1.42 | 0.013997 | PCDH7 | 1.23 | 0.031875 |
| CEPT1 | 2.61 | 2.20E-05 | A1BG | 1.42 | 0.013997 | SMIM18 | 1.23 | 0.031875 |
| CSNK2A2 | 2.58 | 2.70E-05 | FUS | 1.42 | 0.013997 | ADAD2 | -1.23 | 0.031875 |
| FAM231B | -2.54 | 3.20E-05 | SPOPL | 1.42 | 0.013997 | FRY | -1.23 | 0.031875 |
| CCDC101 | 2.47 | 4.40E-05 | FAM159A | -1.42 | 0.013997 | FAM129A | -1.23 | 0.031875 |
| BAP1 | 2.6 | 5.60E-05 | TSC2 | -1.42 | 0.013997 | RAB36 | -1.23 | 0.031875 |
| BCOR | -2.43 | 6.10E-05 | PGAM2 | -1.42 | 0.013997 | RGS12 | -1.23 | 0.031875 |
| PCGF1 | -2.43 | 6.20E-05 | UBE2Q2L | 2.04 | 0.014013 | SMG5 | -1.47 | 0.032635 |
| PPP2R2A | 2.42 | 6.50E-05 | NDUFB11 | 1.5 | 0.014051 | GANAB | 1.37 | 0.032999 |
| EPT1 | 2.42 | 6.50E-05 | UNC13B | 1.5 | 0.014052 | RAET1G | -1.37 | 0.032999 |
| SELRC1 | 2.4 | 7.40E-05 | ABCC1 | -1.5 | 0.014052 | MRPL52 | 1.29 | 0.033021 |
| PTDSS1 | 2.33 | 0.000111 | TRAPPC2 | -1.5 | 0.014052 | RUNDC3A | -1.29 | 0.033021 |
| ZNF708 | -2.45 | 0.000125 | DPH2 | 1.41 | 0.014635 | TMEM14B | 1.22 | 0.033205 |
| YWHAE | 2.3 | 0.000131 | SCRN3 | 1.41 | 0.014635 | C7orf31 | 1.22 | 0.033205 |
| ERCC5 | -2.28 | 0.000149 | IRF3 | 1.41 | 0.014635 | STK16 | 1.22 | 0.033205 |
| IBA57 | 2.26 | 0.000167 | STAP2 | 1.41 | 0.014635 | DLK1 | 1.22 | 0.033205 |
| PAG1 | -2.4 | 0.000169 | KRTAP5-10 | 1.41 | 0.014635 | CST11 | 1.22 | 0.033205 |
| TAF5L | 2.37 | 0.000195 | MFAP4 | -1.41 | 0.014635 | IGFLR1 | 1.22 | 0.033205 |
| ZNF451 | -2.23 | 0.000201 | ATF7IP | -1.41 | 0.014635 | FASTKD1 | 1.22 | 0.033205 |
| PPHLN1 | -2.22 | 0.000214 | SNX27 | -1.41 | 0.014635 | RAB17 | 1.22 | 0.033205 |
| MCM8 | -2.21 | 0.000225 | DMC1 | -1.41 | 0.014635 | FUT3 | 1.22 | 0.033205 |
| BRD1 | -2.2 | 0.000237 | CTNNBL1 | -1.41 | 0.014635 | DBF4B | 1.22 | 0.033205 |
| TAF6L | 2.19 | 0.000252 | TJP3 | -1.41 | 0.014635 | MORC3 | 1.22 | 0.033205 |
| CCDC111 | -2.19 | 0.000252 | TRPV5 | -1.41 | 0.014635 | C22orf24 | 1.22 | 0.033205 |
| ID3 | 2.31 | 0.00027 | SLC24A2 | 1.49 | 0.014671 | ATP6V1C2 | 1.22 | 0.033205 |
| KIAA0586 | 2.17 | 0.000279 | C21orf58 | 1.49 | 0.014671 | ZNF846 | 1.22 | 0.033205 |
| SCAI | -2.14 | 0.000333 | NDUFA12 | 1.49 | 0.014671 | KIAA0895L | -1.22 | 0.033205 |
| SRM | 2.13 | 0.000351 | PCDHGB4 | 1.49 | 0.014671 | MTERF | -1.22 | 0.033205 |
| ADAM12 | 2.13 | 0.000351 | GRIA3 | 1.49 | 0.014671 | SERINC4 | -1.22 | 0.033205 |
| PRPS1 | 2.26 | 0.000355 | CYP4F22 | 1.48 | 0.015306 | AMN | -1.22 | 0.033205 |
| GRHL2 | 2.12 | 0.000373 | C20orf112 | -1.48 | 0.015306 | KCNK16 | -1.22 | 0.033205 |
| BACH2 | -2.24 | 0.000399 | DLX3 | -1.48 | 0.015306 | ABCC5 | -1.22 | 0.033205 |
| VPS33A | -2.1 | 0.000422 | EDNRA | 1.4 | 0.015338 | SNAI1 | -1.22 | 0.033205 |
| ZFY | 2.1 | 0.000423 | CEP76 | 1.4 | 0.015338 | TNFRSF10D | -1.22 | 0.033205 |
| PPP1CC | -2.09 | 0.000447 | TMBIM1 | 1.4 | 0.015338 | UBE2H | -1.22 | 0.033205 |
| TRMT10A | 2.08 | 0.000472 | CD302 | 1.4 | 0.015338 | OAZ2 | -1.22 | 0.033205 |
| AMIGO2 | 2.08 | 0.000473 | CRLS1 | 1.4 | 0.015338 | LCE2A | -1.46 | 0.033714 |
| RNMT | -2.2 | 0.000488 | ANXA11 | -1.4 | 0.015338 | LMAN1L | -1.36 | 0.034224 |
| CARS2 | 2.07 | 0.000499 | SEPT9 | -1.4 | 0.015338 | AKR1C1 | 1.36 | 0.034225 |
| OSR2 | -2.19 | 0.000516 | ZNF614 | -1.4 | 0.015338 | S100G | 1.36 | 0.034225 |
| CSNK1G1 | -2.05 | 0.000564 | ERVMER34-1 | -1.4 | 0.015338 | POU2F1 | -1.36 | 0.034225 |
| GRIPAP1 | 2.04 | 0.000597 | AMPD2 | -1.4 | 0.015338 | GMPR | 1.28 | 0.034313 |
| MBTPS1 | 2.31 | 0.000599 | RBM5 | -1.4 | 0.015338 | MUL1 | -1.28 | 0.034313 |
| LRRC52 | -2.03 | 0.000628 | CUX2 | 1.57 | 0.015449 | C9orf170 | -1.28 | 0.034313 |
| DUSP14 | 2.02 | 0.000665 | TRIM72 | -1.57 | 0.015449 | INPP4B | -1.28 | 0.034313 |
| CMAS | 2.02 | 0.000666 | TRIM44 | -1.47 | 0.015955 | COL4A2 | -1.28 | 0.034313 |
| PIK3R2 | -2.01 | 0.000705 | NEUROD1 | -1.47 | 0.015955 | CP | 1.21 | 0.034612 |
| NDUFA5 | 1.99 | 0.000788 | OR7A5 | -1.47 | 0.015955 | SPATA24 | 1.21 | 0.034612 |
| TXNL4B | -1.98 | 0.000833 | FBXW9 | 1.47 | 0.015956 | LHX1 | 1.21 | 0.034612 |
| GPX5 | 1.97 | 0.000882 | DBF4 | -1.47 | 0.015956 | DEFB132 | 1.21 | 0.034612 |
| TCF3 | -2.23 | 0.000886 | EIF3L | -1.47 | 0.015956 | C21orf2 | -1.21 | 0.034612 |
| NDUFAF3 | 1.96 | 0.000929 | OR6P1 | 1.39 | 0.016017 | CARD8 | -1.21 | 0.034612 |
| CLSTN1 | 2.07 | 0.00095 | FAM127A | 1.39 | 0.016017 | MYO16 | -1.21 | 0.034612 |
| BAK1 | 1.95 | 0.00098 | PPAPDC1B | 1.39 | 0.016017 | GPR1 | -1.21 | 0.034612 |
| SP3 | 2.06 | 0.001002 | CALCOCO1 | 1.39 | 0.016017 | MYSM1 | -1.21 | 0.034612 |
| COQ5 | 1.94 | 0.001038 | C1QBP | 1.39 | 0.016017 | PSIP1 | -1.21 | 0.034612 |
| NDUFB10 | 1.94 | 0.001038 | RLN1 | 1.39 | 0.016017 | RAB7A | -1.21 | 0.034612 |
| RANBP1 | -1.94 | 0.001039 | XRCC3 | -1.39 | 0.016017 | MYO3A | -1.21 | 0.034612 |
| RIF1 | -2.05 | 0.001055 | MTL5 | -1.39 | 0.016017 | SUPT20HL2 | -1.21 | 0.034612 |
| NUDT5 | -2.05 | 0.001056 | ACTB | -1.39 | 0.016017 | CRISP2 | 1.21 | 0.034613 |
| TSSC1 | -1.93 | 0.001093 | TMEM173 | -1.39 | 0.016017 | RAB23 | 1.21 | 0.034613 |
| CREB1 | -1.93 | 0.001093 | CPN1 | -1.39 | 0.016017 | HRAS | 1.21 | 0.034613 |
| METTL17 | 2.04 | 0.001112 | ASPH | -1.39 | 0.016017 | KRT40 | -1.21 | 0.034613 |
| PGP | 2.04 | 0.001112 | PSMB11 | -1.39 | 0.016017 | DMKN | -1.21 | 0.034613 |
| PRRC1 | -2.18 | 0.001123 | CDH18 | -1.39 | 0.016018 | WFDC9 | -1.21 | 0.034613 |
| STRIP2 | 1.92 | 0.001145 | NUDC | 1.56 | 0.016066 | ZNF837 | -1.21 | 0.034613 |
| SHMT2 | 1.92 | 0.001145 | MED25 | 1.56 | 0.016067 | ADIPOR1 | -1.21 | 0.034613 |
| EIF2B4 | -2.03 | 0.001163 | VPS51 | -1.56 | 0.016067 | HDAC4 | -1.21 | 0.034613 |
| MC5R | -1.91 | 0.001209 | ISLR | 1.46 | 0.016637 | C9 | -1.21 | 0.034613 |
| ARL1 | -1.91 | 0.001209 | EDF1 | -1.46 | 0.016637 | ADAMTSL3 | -1.21 | 0.034613 |
| SIPA1L2 | 1.91 | 0.00121 | C11orf63 | -1.46 | 0.016637 | MCM3 | -1.35 | 0.035487 |
| AHSA1 | 1.91 | 0.00121 | GPN2 | -1.46 | 0.016637 | DPP7 | -1.27 | 0.035674 |
| SNRPD1 | -2.32 | 0.001211 | CDK9 | -1.66 | 0.016743 | CPSF3L | -1.27 | 0.035674 |
| RUNX1 | 2.02 | 0.001223 | YARS2 | 1.38 | 0.016748 | FAM73B | 1.27 | 0.035675 |
| UBAP2L | 1.9 | 0.001277 | UHRF2 | 1.38 | 0.016748 | E2F4 | 1.27 | 0.035675 |
| YPEL5 | -1.9 | 0.001277 | TSPAN7 | 1.38 | 0.016748 | MRPS22 | 1.27 | 0.035675 |
| CSK | -1.9 | 0.001277 | PRKCDBP | 1.38 | 0.016748 | LSG1 | 1.27 | 0.035675 |
| GNPNAT1 | 1.89 | 0.001343 | TSPAN12 | -1.38 | 0.016748 | NKG7 | -1.27 | 0.035675 |
| CNOT8 | -1.89 | 0.001343 | GPR20 | -1.38 | 0.016748 | DDX55 | -1.27 | 0.035675 |
| MYEOV | 2 | 0.001355 | IQGAP1 | -1.38 | 0.016748 | CLCA4 | -1.27 | 0.035675 |
| LBX2 | -2 | 0.001355 | CHRNB3 | -1.38 | 0.016748 | MVD | -1.27 | 0.035675 |
| TSNARE1 | 1.88 | 0.001415 | ZNF598 | -1.38 | 0.016748 | ZNF616 | 1.2 | 0.036059 |
| KMT2D | -1.88 | 0.001416 | ARHGAP21 | -1.38 | 0.016748 | MYCBP | 1.2 | 0.036059 |
| NIPSNAP3A | 1.87 | 0.001498 | HN1 | -1.38 | 0.016748 | TAS2R42 | 1.2 | 0.036059 |
| FANCA | -1.87 | 0.001499 | UVSSA | -1.38 | 0.016748 | VCL | 1.2 | 0.036059 |
| PARS2 | 1.86 | 0.001582 | ZNF415 | -1.38 | 0.016748 | GPT2 | 1.2 | 0.036059 |
| CMSS1 | -1.86 | 0.001583 | CYP39A1 | -1.38 | 0.016748 | C9orf64 | 1.2 | 0.036059 |
| GMNN | -1.86 | 0.001583 | EHF | -1.38 | 0.016748 | ACO1 | 1.2 | 0.036059 |
| ITPA | -1.86 | 0.001583 | VPS36 | -1.38 | 0.016748 | GPR124 | 1.2 | 0.036059 |
| BMP2K | 1.84 | 0.001758 | ZNF445 | 1.45 | 0.017346 | TAAR1 | 1.2 | 0.036059 |
| FLRT3 | -1.84 | 0.001758 | GIGYF2 | -1.45 | 0.017346 | ZNF551 | 1.2 | 0.036059 |
| POLG | 1.94 | 0.001838 | KRTCAP2 | -1.45 | 0.017347 | ZSCAN23 | 1.2 | 0.036059 |
| PPP3CA | 1.83 | 0.001865 | PLA2R1 | -1.45 | 0.017347 | CAPN11 | 1.2 | 0.036059 |
| DERA | 1.83 | 0.001866 | ARHGEF38 | -1.45 | 0.017347 | KRT15 | -1.2 | 0.036059 |
| ZNF536 | 1.83 | 0.001866 | NEFM | -1.45 | 0.017347 | MS4A12 | -1.2 | 0.036059 |
| SEC14L6 | -1.83 | 0.001866 | KRTAP22-2 | 1.37 | 0.017522 | UBE2D3 | -1.2 | 0.036059 |
| IFIT2 | -1.83 | 0.001866 | BABAM1 | 1.37 | 0.017522 | TSHZ1 | -1.2 | 0.036059 |
| UBE3D | -1.82 | 0.001971 | IL15 | 1.37 | 0.017522 | TAF1C | -1.2 | 0.036059 |
| DCUN1D2 | -1.82 | 0.001972 | BPIFA2 | -1.37 | 0.017522 | TMEM186 | -1.2 | 0.036059 |
| ZNF430 | -1.82 | 0.001972 | CCDC159 | -1.37 | 0.017522 | APITD1 | -1.2 | 0.036059 |
| GUK1 | -2.05 | 0.002033 | DCLRE1C | -1.37 | 0.017522 | PINK1 | -1.2 | 0.036059 |
| PHTF2 | -1.81 | 0.002076 | YIPF6 | 1.37 | 0.017523 | TAF2 | 1.34 | 0.036798 |
| CCNH | -1.81 | 0.002077 | OR10A6 | 1.37 | 0.017523 | CKS2 | 1.26 | 0.037076 |
| TEX29 | -1.91 | 0.002132 | SAMD12 | 1.37 | 0.017523 | FKBP2 | -1.26 | 0.037076 |
| RCC2 | -1.8 | 0.002193 | TLX3 | -1.37 | 0.017523 | SSU72 | -1.26 | 0.037076 |
| YLPM1 | -2.02 | 0.002312 | ZNF565 | -1.37 | 0.017523 | CCDC158 | -1.26 | 0.037076 |
| TRIAP1 | 1.89 | 0.002344 | HIP1R | -1.37 | 0.017523 | TTC5 | 1.19 | 0.03756 |
| LDB1 | 1.78 | 0.002439 | POLR2L | -1.78 | 0.017807 | MPL | 1.19 | 0.03756 |
| ARL4C | 1.78 | 0.002439 | C4orf40 | 1.44 | 0.018071 | NIPA1 | 1.19 | 0.03756 |
| DRD2 | -1.78 | 0.002439 | SEPSECS | 1.53 | 0.018074 | RFWD2 | -1.19 | 0.03756 |
| STK39 | 1.78 | 0.00244 | TM9SF2 | 1.36 | 0.018346 | TRIM28 | 1.19 | 0.037561 |
| PADI6 | 1.87 | 0.002599 | TADA2B | 1.36 | 0.018346 | WNT5A | 1.19 | 0.037561 |
| WNT10B | 1.76 | 0.002707 | RTP2 | 1.36 | 0.018346 | NDUFS3 | 1.19 | 0.037561 |
| LYSMD1 | 1.76 | 0.002707 | ASMT | 1.36 | 0.018346 | PITX2 | 1.19 | 0.037561 |
| RWDD1 | -1.76 | 0.002707 | SH3GL2 | 1.36 | 0.018346 | SFXN4 | 1.19 | 0.037561 |
| PVRL1 | 1.86 | 0.002729 | WDYHV1 | -1.36 | 0.018346 | SDCBP2 | 1.19 | 0.037561 |
| AASDHPPT | 1.75 | 0.002848 | SCGB1A1 | -1.36 | 0.018346 | GABARAPL2 | 1.19 | 0.037561 |
| MOBP | 1.75 | 0.002849 | EIF2A | -1.36 | 0.018346 | OR1M1 | 1.19 | 0.037561 |
| GTPBP2 | -1.85 | 0.002861 | GPA33 | -1.36 | 0.018346 | KIF1C | 1.19 | 0.037561 |
| RBFA | 1.74 | 0.002996 | GSS | 1.36 | 0.018347 | GZMA | 1.19 | 0.037561 |
| CATSPER4 | 1.74 | 0.002996 | CXCL5 | 1.36 | 0.018347 | MYO1C | 1.19 | 0.037561 |
| DIXDC1 | 1.84 | 0.003002 | EML6 | -1.36 | 0.018347 | LRRC1 | 1.19 | 0.037561 |
| PAPD5 | 1.84 | 0.003003 | RPL11 | 1.77 | 0.018402 | NDST3 | 1.19 | 0.037561 |
| TMPO | -1.73 | 0.003154 | PNPT1 | 1.52 | 0.018796 | GALNT9 | -1.19 | 0.037561 |
| PCDHB5 | -1.73 | 0.003155 | SYS1 | -1.52 | 0.018796 | C12orf44 | -1.19 | 0.037561 |
| DCAF11 | 1.83 | 0.003164 | ACOT11 | 1.43 | 0.018826 | MEF2BNB | -1.19 | 0.037561 |
| ZNF486 | 1.82 | 0.003317 | CA14 | 1.43 | 0.018826 | SLC26A5 | -1.19 | 0.037561 |
| GMEB1 | -1.82 | 0.003317 | NDUFB7 | 1.43 | 0.018826 | IL10 | -1.19 | 0.037561 |
| SEC24D | 1.72 | 0.003324 | CPSF6 | -1.43 | 0.018826 | KLHL21 | -1.19 | 0.037561 |
| C4BPB | 1.72 | 0.003324 | BCAM | 1.43 | 0.018827 | WWP1 | -1.19 | 0.037561 |
| SUPT20H | 1.72 | 0.003324 | DCBLD2 | -1.43 | 0.018827 | TMEM86A | -1.19 | 0.037561 |
| C12orf66 | 1.72 | 0.003324 | GOLGA6D | -1.43 | 0.018827 | QRICH1 | -1.33 | 0.03813 |
| RBM8A | 1.93 | 0.003441 | CXorf40A | 1.35 | 0.019158 | EPHA7 | 1.25 | 0.038524 |
| MLST8 | 1.71 | 0.00349 | ZNF639 | -1.35 | 0.019158 | ADAM22 | 1.25 | 0.038524 |
| NUDCD1 | 1.71 | 0.00349 | CD14 | -1.35 | 0.019158 | TMEM82 | 1.25 | 0.038524 |
| NARS2 | 1.71 | 0.00349 | ZEB1 | 1.35 | 0.019159 | KRTAP5-6 | 1.25 | 0.038524 |
| ICT1 | 1.71 | 0.00349 | SCN8A | 1.35 | 0.019159 | DOLK | 1.25 | 0.038524 |
| NKTR | 2.06 | 0.0036 | TIGD7 | 1.35 | 0.019159 | ZPBP | 1.25 | 0.038524 |
| S100A13 | 1.7 | 0.00367 | SLFN11 | 1.35 | 0.019159 | SRF | -1.25 | 0.038524 |
| SCO2 | 1.7 | 0.00367 | GNB5 | 1.35 | 0.019159 | ATF6 | 1.25 | 0.038525 |
| KDM6B | 1.7 | 0.00367 | GDPD1 | 1.35 | 0.019159 | SYNPR | 1.18 | 0.039092 |
| CAMK2G | -1.7 | 0.00367 | UGT3A1 | -1.35 | 0.019159 | LRRC14 | 1.18 | 0.039092 |
| DARS2 | 1.91 | 0.003748 | KCNJ13 | -1.35 | 0.019159 | DYX1C1 | -1.18 | 0.039092 |
| RSRC2 | -2.22 | 0.003787 | EIF4ENIF1 | -1.35 | 0.019159 | OR2A1 | -1.18 | 0.039092 |
| ANKRD17 | -1.79 | 0.003826 | ZBTB7A | -1.35 | 0.019159 | RASL10B | -1.18 | 0.039092 |
| TCF12 | -1.79 | 0.003826 | VPS25 | -1.93 | 0.01944 | CAMK1 | -1.18 | 0.039092 |
| BPTF | 1.79 | 0.003827 | AZI1 | -1.51 | 0.019515 | CWF19L1 | 1.18 | 0.039093 |
| KPTN | 1.69 | 0.00385 | NRBP1 | -1.51 | 0.019516 | DAP3 | 1.18 | 0.039093 |
| CDC42EP2 | -1.69 | 0.00385 | DUSP10 | 1.42 | 0.01964 | TCTA | 1.18 | 0.039093 |
| CLTB | -1.78 | 0.004011 | TAB2 | 1.42 | 0.019641 | OMA1 | 1.18 | 0.039093 |
| NANS | 1.68 | 0.004046 | NIPSNAP3B | 1.34 | 0.020006 | SSR3 | 1.18 | 0.039093 |
| VASP | -1.68 | 0.004046 | SLC25A51 | 1.34 | 0.020006 | ATCAY | 1.18 | 0.039093 |
| CLPP | 1.68 | 0.004047 | SCN4B | -1.34 | 0.020006 | CLNK | 1.18 | 0.039093 |
| UBE2N | -1.68 | 0.004047 | FANCC | -1.34 | 0.020006 | ATF4 | 1.18 | 0.039093 |
| CDIPT | -1.77 | 0.004204 | SPATA8 | -1.34 | 0.020006 | TMEM9 | -1.18 | 0.039093 |
| ATP6AP1 | -2.19 | 0.004235 | PSPC1 | -1.34 | 0.020006 | PCBP2 | -1.18 | 0.039093 |
| PMAIP1 | 1.67 | 0.004248 | LYZL4 | -1.34 | 0.020006 | SLC35E1 | -1.18 | 0.039093 |
| VDAC1 | 1.67 | 0.004248 | RFX8 | -1.34 | 0.020006 | COLEC12 | -1.18 | 0.039093 |
| DNLZ | 1.67 | 0.004248 | BNIP2 | 1.34 | 0.020007 | OR2A42 | -1.18 | 0.039093 |
| CST4 | -1.67 | 0.004248 | GPR125 | 1.34 | 0.020007 | CNOT2 | -1.18 | 0.039093 |
| RNASE6 | 1.67 | 0.004249 | PIK3CD | 1.34 | 0.020007 | APITD1 | -1.18 | 0.039093 |
| RHOA | -1.67 | 0.004249 | ZC3H15 | 1.34 | 0.020007 | TOX2 | -1.18 | 0.039093 |
| CALML4 | -1.88 | 0.004271 | FAM163B | -1.61 | 0.020012 | C1orf112 | -1.18 | 0.039093 |
| WDR26 | -1.88 | 0.004272 | WDFY4 | 1.33 | 0.020881 | CCR10 | -1.18 | 0.039093 |
| RTCB | -1.88 | 0.004272 | RAF1 | 1.33 | 0.020881 | TAF1B | -1.18 | 0.039093 |
| KRTAP6-3 | 1.76 | 0.004396 | OR8H1 | -1.33 | 0.020881 | FAM134C | -1.18 | 0.039093 |
| NDUFS1 | 1.87 | 0.004453 | NDUFS6 | 1.33 | 0.020882 | KRT37 | -1.18 | 0.039093 |
| RTTN | -1.66 | 0.004476 | TMEM104 | 1.33 | 0.020882 | ZNF496 | -1.18 | 0.039093 |
| HSF1 | 1.66 | 0.004477 | TMEM99 | 1.33 | 0.020882 | DHDDS | -1.32 | 0.039512 |
| WWC3 | -1.66 | 0.004477 | UXS1 | 1.33 | 0.020882 | TMSB15B | 1.68 | 0.039585 |
| OR2F1 | 1.75 | 0.004602 | DNAJC22 | 1.33 | 0.020882 | TARS | -1.41 | 0.039831 |
| TRIP12 | 1.75 | 0.004602 | SNX7 | 1.33 | 0.020882 | BBC3 | 1.24 | 0.040047 |
| CDK12 | -1.75 | 0.004602 | PTK2B | 1.33 | 0.020882 | CPSF3 | 1.24 | 0.040047 |
| SLC5A3 | -1.65 | 0.004699 | CERS3 | 1.33 | 0.020882 | COL23A1 | 1.24 | 0.040047 |
| EIF4E2 | -1.65 | 0.004699 | RBM45 | 1.33 | 0.020882 | UBA5 | 1.24 | 0.040047 |
| TMEM208 | 1.65 | 0.0047 | HBG1 | -1.33 | 0.020882 | NDUFAF1 | 1.24 | 0.040047 |
| PHF15 | -1.65 | 0.0047 | IZUMO2 | -1.33 | 0.020882 | ASXL2 | 1.24 | 0.040047 |
| TMED10 | 1.74 | 0.004828 | BRCA1 | -1.33 | 0.020882 | GRK5 | -1.24 | 0.040047 |
| MOG | -1.74 | 0.004828 | DLL1 | -1.33 | 0.020882 | PDLIM3 | 1.17 | 0.040704 |
| DDX27 | -1.85 | 0.004853 | BMP6 | 1.4 | 0.021284 | C10orf128 | 1.17 | 0.040704 |
| GJA3 | 1.64 | 0.004938 | FRRS1 | -1.4 | 0.021284 | PDSS1 | 1.17 | 0.040704 |
| PDLIM5 | -1.64 | 0.004939 | DNAJA4 | 1.4 | 0.021285 | MYOT | 1.17 | 0.040704 |
| HEXIM1 | -1.64 | 0.004939 | ZNF202 | 1.4 | 0.021285 | TMEM133 | 1.17 | 0.040704 |
| GLTPD1 | -1.64 | 0.004939 | TRIM31 | 1.32 | 0.021809 | POU3F3 | 1.17 | 0.040704 |
| RNASE9 | -1.64 | 0.004939 | CDK5R1 | 1.32 | 0.021809 | SORD | 1.17 | 0.040704 |
| CCDC36 | 1.63 | 0.005188 | C1orf159 | 1.32 | 0.021809 | OR8H2 | 1.17 | 0.040704 |
| UGT1A9 | -1.63 | 0.005188 | TMEM106B | 1.32 | 0.021809 | EPOR | 1.17 | 0.040704 |
| CDK15 | -1.63 | 0.005188 | FZR1 | -1.32 | 0.021809 | S100A2 | -1.17 | 0.040704 |
| SKP2 | -1.63 | 0.005188 | CALB2 | -1.32 | 0.021809 | PRSS21 | 1.17 | 0.040705 |
| ATP5S | 1.63 | 0.005189 | OR4K5 | 1.32 | 0.02181 | RRNAD1 | 1.17 | 0.040705 |
| RPIA | 1.63 | 0.005189 | NDUFAF4 | 1.32 | 0.02181 | COX16 | 1.17 | 0.040705 |
| LATS2 | 1.63 | 0.005189 | PRKAR1A | 1.32 | 0.02181 | CLDN14 | 1.17 | 0.040705 |
| C6orf136 | -1.63 | 0.005189 | RNF208 | 1.32 | 0.02181 | NRTN | 1.17 | 0.040705 |
| HOMEZ | 1.72 | 0.005297 | THAP2 | 1.32 | 0.02181 | DLG1 | -1.17 | 0.040705 |
| EML2 | 1.72 | 0.005297 | SLC22A9 | -1.32 | 0.02181 | WIPF1 | -1.17 | 0.040705 |
| ODC1 | 1.62 | 0.005455 | ELAVL1 | -1.32 | 0.02181 | LMBRD2 | -1.17 | 0.040705 |
| DNTTIP1 | -1.62 | 0.005455 | NEFH | -1.32 | 0.02181 | GORASP1 | -1.17 | 0.040705 |
| CUBN | -1.62 | 0.005455 | ZNF23 | -1.32 | 0.02181 | FAM160B1 | -1.17 | 0.040705 |
| C3orf30 | 1.62 | 0.005456 | VMA21 | -1.32 | 0.02181 | FGG | -1.17 | 0.040705 |
| ATE1 | -1.62 | 0.005456 | BOC | -1.32 | 0.02181 | TET3 | -1.17 | 0.040705 |
| HPN | 1.71 | 0.005543 | CHD1L | -1.32 | 0.02181 | PRDX1 | -1.17 | 0.040705 |
| MORN2 | 2.11 | 0.005661 | HK2 | 1.39 | 0.022154 | C5orf47 | -1.17 | 0.040705 |
| FASTK | 1.61 | 0.005727 | DDX53 | 1.39 | 0.022154 | PRR25 | -1.17 | 0.040705 |
| SUPT7L | 1.61 | 0.005727 | RECQL5 | -1.39 | 0.022154 | ADSL | -1.4 | 0.041129 |
| MAPK1 | 1.61 | 0.005727 | MAVS | -1.39 | 0.022154 | ALG1L | 1.23 | 0.041559 |
| DDX28 | 1.61 | 0.005727 | MTHFD2 | 1.31 | 0.022792 | OSGEP | -1.23 | 0.041559 |
| TUBB1 | 1.61 | 0.005727 | RAC3 | -1.31 | 0.022792 | SLC8A3 | 1.23 | 0.04156 |
| KCNK18 | -1.61 | 0.005727 | LRRC61 | -1.31 | 0.022792 | ASB3 | 1.23 | 0.04156 |
| CUL4B | -1.61 | 0.005728 | SPIRE1 | 1.31 | 0.022793 | SRRT | -1.23 | 0.04156 |
| OR5I1 | 1.7 | 0.005801 | RGS10 | 1.31 | 0.022793 | ACSL3 | -1.23 | 0.04156 |
| GNA11 | -1.7 | 0.005801 | ZNF330 | 1.31 | 0.022793 | LASP1 | -1.23 | 0.04156 |
| UFM1 | 1.6 | 0.00601 | TMEM161A | 1.31 | 0.022793 | ULK2 | -1.23 | 0.04156 |
| DPEP3 | -1.6 | 0.00601 | UBE2A | 1.31 | 0.022793 | OR4M2 | -1.51 | 0.041839 |
| OTX2 | -1.6 | 0.00601 | CYP4A22 | 1.31 | 0.022793 | RND1 | 1.16 | 0.042387 |
| USP5 | -1.69 | 0.006083 | BANP | -1.31 | 0.022793 | KIF7 | 1.16 | 0.042387 |
| TEX261 | 1.59 | 0.00631 | GPR107 | -1.31 | 0.022793 | ORMDL2 | 1.16 | 0.042387 |
| NGFRAP1 | 1.59 | 0.006311 | RUFY3 | -1.31 | 0.022793 | CECR6 | -1.16 | 0.042387 |
| CTBP1 | -1.59 | 0.006311 | GHITM | -1.31 | 0.022793 | SULF2 | -1.16 | 0.042387 |
| GLI4 | 1.58 | 0.006617 | SLC6A6 | -1.31 | 0.022793 | DENND6B | -1.16 | 0.042387 |
| ZBTB10 | -1.58 | 0.006617 | PDIA5 | -1.31 | 0.022793 | TCEAL7 | -1.16 | 0.042387 |
| TIMM50 | 1.67 | 0.006667 | OR51A4 | -1.31 | 0.022793 | ZNF543 | -1.16 | 0.042387 |
| TARM1 | 1.57 | 0.006924 | MTX1 | 1.38 | 0.023081 | EVX1 | -1.16 | 0.042387 |
| NDUFAF6 | 1.57 | 0.006924 | ZNF217 | -1.38 | 0.023081 | KIAA1755 | -1.16 | 0.042387 |
| TNFSF11 | 1.57 | 0.006924 | HDAC3 | -1.38 | 0.023081 | P2RY12 | -1.16 | 0.042387 |
| MS4A6A | 1.57 | 0.006924 | TXNDC11 | -1.46 | 0.023623 | ARPC4 | -1.16 | 0.042387 |
| ZNF280D | 1.57 | 0.006924 | VAV2 | 1.3 | 0.023786 | OR6C76 | -1.16 | 0.042387 |
| CCNB1 | -1.57 | 0.006924 | LHX9 | 1.3 | 0.023786 | KRTAP5-11 | -1.16 | 0.042387 |
| P3H3 | -1.57 | 0.006924 | CCDC90B | 1.3 | 0.023786 | KDELC2 | -1.16 | 0.042387 |
| SLC2A2 | -1.57 | 0.006924 | MAPKAPK3 | 1.3 | 0.023787 | ATP5SL | 1.16 | 0.042388 |
| SUPT16H | -1.66 | 0.006956 | PET112 | 1.3 | 0.023787 | SLCO1C1 | 1.16 | 0.042388 |
| OSCP1 | 1.56 | 0.007265 | IFIT5 | 1.3 | 0.023787 | C1orf158 | 1.16 | 0.042388 |
| OR4P4 | 1.56 | 0.007265 | CACNG6 | 1.3 | 0.023787 | AK9 | 1.16 | 0.042388 |
| ABHD14A | 1.56 | 0.007265 | RNF168 | -1.3 | 0.023787 | AWAT2 | 1.16 | 0.042388 |
| CENPV | 1.56 | 0.007265 | CLDN15 | -1.3 | 0.023787 | RNF2 | -1.16 | 0.042388 |
| PFDN2 | -1.56 | 0.007265 | ANP32B | -1.3 | 0.023787 | PRDX6 | -1.16 | 0.042388 |
| RIMS2 | -1.56 | 0.007265 | AMOT | -1.3 | 0.023787 | IGFBP6 | -1.16 | 0.042388 |
| LHX2 | -1.56 | 0.007265 | SH3BP5L | -1.3 | 0.023787 | SNX33 | -1.16 | 0.042388 |
| FANCD2 | -1.56 | 0.007265 | WDR43 | -1.56 | 0.023929 | CLEC12A | -1.16 | 0.042388 |
| OR11L1 | -1.56 | 0.007265 | ZKSCAN1 | 1.37 | 0.024026 | MSI2 | -1.16 | 0.042388 |
| ZC3H7A | 1.56 | 0.007266 | CXorf56 | 1.37 | 0.024026 | DPF2 | -1.16 | 0.042388 |
| INTS12 | -1.56 | 0.007266 | NDUFA2 | 1.37 | 0.024026 | SEC16A | 1.3 | 0.042426 |
| DAXX | -1.56 | 0.007266 | C16orf80 | -1.37 | 0.024026 | PPA1 | 1.3 | 0.042426 |
| TICAM1 | -1.65 | 0.007277 | ROMO1 | 1.37 | 0.024027 | ZWINT | -1.3 | 0.042426 |
| CHD8 | -1.65 | 0.007277 | TACR3 | -1.37 | 0.024027 | C1orf35 | -1.3 | 0.042426 |
| ZNF211 | 1.55 | 0.007622 | TIMP4 | 1.29 | 0.02482 | MARCH2 | 1.22 | 0.043173 |
| PTPN13 | 1.55 | 0.007622 | AUNIP | 1.29 | 0.02482 | PEX13 | -1.22 | 0.043173 |
| QKI | -1.55 | 0.007622 | CBLN3 | 1.29 | 0.02482 | PPIH | 1.22 | 0.043174 |
| FAM161B | 1.55 | 0.007623 | MXRA8 | 1.29 | 0.02482 | BTBD19 | 1.22 | 0.043174 |
| WDR70 | -1.55 | 0.007623 | NEFL | 1.29 | 0.02482 | TFPT | -1.22 | 0.043174 |
| OTUB1 | -1.64 | 0.007625 | SEC63 | 1.29 | 0.02482 | SENP1 | -1.22 | 0.043174 |
| ATP5D | 1.63 | 0.007977 | FBLN1 | 1.29 | 0.02482 | IGF1R | -1.22 | 0.043174 |
| TCOF1 | -1.63 | 0.007977 | CXorf27 | 1.29 | 0.02482 | CDC42EP4 | 1.29 | 0.043931 |
| AICDA | -1.63 | 0.007977 | SSR2 | 1.29 | 0.02482 | THY1 | 1.29 | 0.043931 |
| L2HGDH | 1.54 | 0.008002 | XPNPEP3 | 1.29 | 0.02482 | UNC50 | 1.15 | 0.044093 |
| ARNTL2 | 1.54 | 0.008003 | FAM19A1 | 1.29 | 0.02482 | MRPL21 | 1.15 | 0.044093 |
| R3HDM4 | -1.54 | 0.008003 | SNTA1 | 1.29 | 0.02482 | RNF19A | 1.15 | 0.044093 |
| CYSLTR2 | -1.54 | 0.008003 | KIF18A | 1.29 | 0.02482 | GPC5 | 1.15 | 0.044093 |
| PICALM | -1.54 | 0.008003 | C17orf66 | 1.29 | 0.02482 | QRSL1 | 1.15 | 0.044093 |
| NELFB | -1.73 | 0.008066 | FBXO30 | -1.29 | 0.02482 | CDC42BPA | 1.15 | 0.044093 |
| MTIF3 | 1.62 | 0.008331 | PPP1R26 | -1.29 | 0.02482 | TUBE1 | 1.15 | 0.044093 |
| PPP1R37 | 1.62 | 0.008332 | COL2A1 | -1.29 | 0.02482 | ICOSLG | -1.15 | 0.044093 |
| MCM9 | -1.62 | 0.008332 | C10orf120 | -1.29 | 0.02482 | SNCA | -1.15 | 0.044093 |
| RPL36 | 2.49 | 0.008359 | HIST2H2AC | 1.36 | 0.025021 | FANCF | -1.15 | 0.044093 |
| ARL2BP | 1.53 | 0.008387 | FSIP2 | -1.36 | 0.025021 | MCTS1 | -1.15 | 0.044093 |
| UBE3A | 1.53 | 0.008387 | PIP4K2B | 1.44 | 0.025473 | RBAK | -1.15 | 0.044093 |
| ZKSCAN7 | 1.53 | 0.008387 | N6AMT1 | 1.28 | 0.025912 | MCAT | 1.15 | 0.044094 |
| CYP2S1 | 1.53 | 0.008387 | FAM19A4 | 1.28 | 0.025912 | C4A | 1.15 | 0.044094 |
| NGLY1 | 1.53 | 0.008387 | GNPDA1 | 1.28 | 0.025912 | FBXO47 | 1.15 | 0.044094 |
| THEG | -1.53 | 0.008387 | KCNAB3 | 1.28 | 0.025912 | PEX2 | 1.15 | 0.044094 |
| CNTD1 | -1.53 | 0.008387 | GUF1 | 1.28 | 0.025912 | KRT33A | 1.15 | 0.044094 |
| PNPO | -1.61 | 0.0087 | MROH9 | -1.28 | 0.025912 | ARHGDIB | 1.15 | 0.044094 |
| NEMF | -1.61 | 0.008701 | ING1 | -1.28 | 0.025912 | AMY2B | 1.15 | 0.044094 |
| GALR2 | -1.52 | 0.008789 | STK40 | -1.28 | 0.025912 | CELF1 | 1.15 | 0.044094 |
| LRRC47 | 1.52 | 0.00879 | HRNR | -1.28 | 0.025912 | ZNF568 | 1.15 | 0.044094 |
| ITGB3BP | -1.6 | 0.00909 | UTP11L | -1.28 | 0.025912 | CHI3L2 | 1.15 | 0.044094 |
| CLUH | 1.6 | 0.009091 | COL16A1 | 1.28 | 0.025913 | LYPD2 | 1.15 | 0.044094 |
| EZH1 | 1.7 | 0.00914 | NDUFB2 | 1.28 | 0.025913 | NCS1 | 1.15 | 0.044094 |
| SLX4 | -1.51 | 0.009227 | PREPL | 1.28 | 0.025913 | SERTAD4 | 1.15 | 0.044094 |
| ZBTB44 | 1.51 | 0.009228 | ABL1 | 1.28 | 0.025913 | C10orf95 | -1.15 | 0.044094 |
| RSPH3 | 1.59 | 0.009507 | CPNE8 | 1.28 | 0.025913 | NRD1 | -1.15 | 0.044094 |
| RBMX | -1.69 | 0.009532 | DSCR4 | -1.28 | 0.025913 | RXRB | -1.15 | 0.044094 |
| SYT12 | 1.5 | 0.009688 | ZNF517 | -1.28 | 0.025913 | KIAA1432 | -1.15 | 0.044094 |
| THAP9 | 1.5 | 0.009688 | RPL3 | -1.28 | 0.025913 | UGT8 | -1.15 | 0.044094 |
| VEZT | 1.5 | 0.009688 | MTF2 | 1.35 | 0.026025 | KLHL31 | -1.15 | 0.044094 |
| LIPK | 1.5 | 0.009688 | UBE2G2 | 1.35 | 0.026025 | PTCHD1 | -1.15 | 0.044094 |
| FBXW5 | 1.5 | 0.009688 | NOL9 | -1.35 | 0.026025 | C9orf135 | 1.21 | 0.044812 |
| TESC | 1.5 | 0.009688 | ST8SIA6 | -1.35 | 0.026025 | FNBP4 | 1.21 | 0.044812 |
| TRIM77 | 1.5 | 0.009688 | PQLC3 | -1.35 | 0.026025 | TXNRD3 | 1.21 | 0.044812 |
| FCHO1 | 1.5 | 0.009688 | RRM2 | -2.05 | 0.026352 | DCAF8 | 1.21 | 0.044812 |
| NDUFB5 | 1.5 | 0.009688 | OR4N5 | 1.27 | 0.027022 | BAG4 | 1.21 | 0.044812 |
| UGP2 | 1.5 | 0.009688 | DCUN1D5 | -1.27 | 0.027022 | MARVELD3 | -1.21 | 0.044812 |
| TNFAIP3 | -1.5 | 0.009688 | ZNF655 | 1.27 | 0.027023 | PLRG1 | -1.21 | 0.044812 |
| NFRKB | -1.5 | 0.009688 | RALGPS1 | 1.27 | 0.027023 | C2orf70 | -1.21 | 0.044812 |
| CELF3 | -1.5 | 0.009688 | CREBRF | 1.27 | 0.027023 | POLR2B | 1.37 | 0.045403 |
| CHMP7 | -1.5 | 0.009688 | ID4 | 1.27 | 0.027023 | MSR1 | 1.28 | 0.045489 |
| KIF11 | -2.16 | 0.009716 | PAGR1 | 1.27 | 0.027023 | LRP10 | -1.28 | 0.045489 |
| RPS13 | -1.8 | 0.009927 | SNAPIN | 1.27 | 0.027023 | POLRMT | 1.14 | 0.0459 |
| AHCYL1 | 1.58 | 0.009949 | LACE1 | 1.27 | 0.027023 | PRSS37 | 1.14 | 0.0459 |
| DNAJC9 | -1.58 | 0.009949 | ANGPTL5 | 1.27 | 0.027023 | CPT2 | 1.14 | 0.0459 |
| ZNF461 | -1.58 | 0.009949 | NETO2 | 1.27 | 0.027023 | ILVBL | 1.14 | 0.0459 |
| TXNIP | 1.49 | 0.010147 | COPS4 | 1.27 | 0.027023 | PHF19 | 1.14 | 0.0459 |
| SOST | 1.49 | 0.010147 | GPR151 | 1.27 | 0.027023 | NTNG1 | 1.14 | 0.0459 |
| TNFRSF13C | 1.49 | 0.010148 | BTG4 | -1.27 | 0.027023 | MBIP | 1.14 | 0.0459 |
| NDUFB1 | 1.49 | 0.010148 | NOC4L | -1.27 | 0.027023 | PSKH2 | 1.14 | 0.0459 |
| CCNT1 | 1.49 | 0.010148 | MMP3 | -1.27 | 0.027023 | HERPUD2 | 1.14 | 0.0459 |
| PINX1 | -1.49 | 0.010148 | LCE5A | -1.27 | 0.027023 | WRN | -1.14 | 0.0459 |
| TGFBR3 | 1.57 | 0.010388 | TMEM160 | -1.27 | 0.027023 | NUCB2 | -1.14 | 0.0459 |
| FHOD3 | 1.57 | 0.010388 | POLR3K | 1.34 | 0.027087 | FAM3C | -1.14 | 0.0459 |
| GNAS | 1.48 | 0.010628 | CCDC33 | 1.34 | 0.027087 | ZNF689 | -1.14 | 0.0459 |
| LBX1 | -1.48 | 0.010628 | CTDSPL2 | 1.34 | 0.027087 | ALKBH8 | -1.14 | 0.0459 |
| ATRX | -1.48 | 0.010628 | CAMKV | 1.34 | 0.027088 | PTPRC | -1.14 | 0.0459 |
| WDR1 | -1.48 | 0.010628 | CD82 | 1.34 | 0.027088 | PCDHB2 | -1.14 | 0.0459 |
| HERC6 | -1.48 | 0.010628 | TFDP1 | -1.34 | 0.027088 | NPFFR2 | 1.14 | 0.045901 |
| CNST | 1.48 | 0.010629 | EGR2 | -1.42 | 0.027459 | C17orf75 | 1.14 | 0.045901 |
| PIGN | 1.48 | 0.010629 | DACT1 | 1.26 | 0.02814 | TCF24 | -1.14 | 0.045901 |
| KLHL14 | -1.48 | 0.010629 | CCDC178 | 1.26 | 0.02814 | DOCK7 | -1.14 | 0.045901 |
| TUBB3 | -1.66 | 0.010794 | ZNF41 | 1.26 | 0.02814 | KIR3DL3 | -1.14 | 0.045901 |
| HTR3D | -1.56 | 0.010859 | ENOX1 | 1.26 | 0.02814 | ITPKB | 1.2 | 0.046543 |
| SACS | 1.56 | 0.01086 | DNAAF1 | -1.26 | 0.02814 | RALY | 1.2 | 0.046543 |
| MAL | 1.56 | 0.01086 | AGPAT3 | -1.26 | 0.02814 | PHF16 | 1.2 | 0.046543 |
| ZSCAN20 | -1.56 | 0.01086 | CCDC59 | -1.26 | 0.02814 | MYC | 1.62 | 0.046543 |
| SMARCD1 | 1.47 | 0.011131 | CYP2E1 | -1.26 | 0.02814 | WTAP | -1.2 | 0.046543 |
| NUBPL | 1.47 | 0.011131 | GLP1R | -1.26 | 0.02814 | ETV3L | -1.2 | 0.046543 |
| ORM2 | -1.47 | 0.011131 | PNKD | -1.26 | 0.02814 | SOAT1 | 1.2 | 0.046544 |
| RDX | -1.47 | 0.011131 | PNRC2 | -1.26 | 0.02814 | CLP1 | 1.36 | 0.04693 |
| HBG2 | -1.47 | 0.011131 | ZNF292 | -1.26 | 0.02814 | DDX6 | -1.27 | 0.047119 |
| EDC4 | -1.47 | 0.011131 | CORO1C | 1.26 | 0.028141 | ECSIT | 1.13 | 0.047761 |
| CAPRIN2 | -1.47 | 0.011131 | GPR68 | 1.26 | 0.028141 | KRTAP19-1 | 1.13 | 0.047761 |
| KCNE4 | 1.47 | 0.011132 | ATP8B4 | 1.26 | 0.028141 | IFNA10 | 1.13 | 0.047761 |
| TBC1D10C | 1.47 | 0.011132 | HOOK2 | -1.26 | 0.028141 | GFOD1 | 1.13 | 0.047761 |
| CKB | -1.47 | 0.011132 | NDUFAF5 | 1.41 | 0.028518 | CCNL1 | 1.13 | 0.047761 |
| F3 | 1.55 | 0.011357 | RARS | 1.63 | 0.028796 | CCNG1 | 1.13 | 0.047761 |
| ZNF831 | 1.55 | 0.011357 | MRGPRE | 1.32 | 0.029323 | NKX2-5 | 1.13 | 0.047761 |
| SETD1B | -1.55 | 0.011357 | MEIS1 | 1.25 | 0.029324 | CES4A | 1.13 | 0.047761 |
| KRTAP4-6 | -1.55 | 0.011357 | IL27RA | 1.25 | 0.029324 | WDR72 | 1.13 | 0.047761 |
| FAM183A | 1.55 | 0.011358 | ASXL1 | 1.25 | 0.029324 | NPAS2 | 1.13 | 0.047761 |
| GPI | 1.91 | 0.011467 | FBXW8 | 1.25 | 0.029324 | WFDC11 | 1.13 | 0.047761 |
| PHF5A | 1.76 | 0.011572 | ADI1 | 1.25 | 0.029324 | PCDHB3 | 1.13 | 0.047761 |
| C15orf27 | -1.46 | 0.011644 | ALG10 | 1.25 | 0.029324 | RAB6B | 1.13 | 0.047761 |
| B9D2 | 1.46 | 0.011645 | ELF1 | 1.25 | 0.029324 | ARHGEF19 | 1.13 | 0.047761 |
| WNT3 | -1.46 | 0.011645 | ZDHHC7 | 1.25 | 0.029324 | APC2 | -1.13 | 0.047761 |
| ANP32A | -1.46 | 0.011645 | PPP2R3C | 1.25 | 0.029324 | C1orf195 | -1.13 | 0.047761 |
| ANKEF1 | 1.54 | 0.011854 | SGCZ | 1.25 | 0.029324 | DNAJC13 | -1.13 | 0.047761 |
| FAF2 | 1.54 | 0.011854 | OSBPL10 | 1.25 | 0.029324 | PCDHB12 | -1.13 | 0.047761 |
| DGAT1 | 1.54 | 0.011854 | KIF3A | 1.25 | 0.029324 | SLC25A39 | -1.13 | 0.047761 |
| STRADB | 1.54 | 0.011854 | KDM5C | 1.32 | 0.029324 | TVP23C | -1.13 | 0.047761 |
| CCDC96 | -1.54 | 0.011854 | SCLY | 1.32 | 0.029324 | MFSD5 | -1.13 | 0.047761 |
| ETFDH | 1.54 | 0.011855 | LOC81691 | -1.32 | 0.029324 | ASIC1 | -1.13 | 0.047761 |
| SHB | -1.63 | 0.01217 | SPARC | -1.25 | 0.029324 | PRRC2A | -1.13 | 0.047761 |
| TUBG1 | -1.63 | 0.01217 | MAPK4 | -1.25 | 0.029324 | ZNF669 | -1.13 | 0.047761 |
| CNOT7 | -1.45 | 0.012192 | SDHD | -1.25 | 0.029324 | DYRK4 | -1.13 | 0.047761 |
| GCKR | 1.45 | 0.012193 | SUV39H2 | -1.25 | 0.029324 | HEATR3 | 1.13 | 0.047762 |
| ITM2C | 1.45 | 0.012193 | FAM65A | -1.25 | 0.029324 | NT5C1A | 1.13 | 0.047762 |
| MTHFD1L | 1.45 | 0.012193 | FNDC9 | -1.25 | 0.029324 | VIPR1 | -1.13 | 0.047762 |
| CLPB | 1.45 | 0.012193 | WNT4 | -1.25 | 0.029324 | B4GALT3 | -1.13 | 0.047762 |
| MTHFR | 1.45 | 0.012193 | T | -1.25 | 0.029324 | RRP9 | -1.13 | 0.047762 |
| COL27A1 | -1.45 | 0.012193 | ADAM29 | -1.25 | 0.029324 | TRIM22 | -1.13 | 0.047762 |
| DFNB59 | -1.45 | 0.012193 | CALM3 | -1.25 | 0.029324 | CHD5 | -1.13 | 0.047762 |
| TPRX1 | -1.45 | 0.012193 | ENO2 | -1.25 | 0.029324 | PLK2 | 1.19 | 0.048316 |
| SSH2 | -1.53 | 0.012366 | ADRA2A | -1.25 | 0.029324 | OR52N4 | 1.19 | 0.048316 |
| COPS2 | -1.53 | 0.012367 | PLCH1 | -1.25 | 0.029324 | BTAF1 | 1.19 | 0.048316 |
| ALYREF | -1.53 | 0.012367 | COX7B | 1.31 | 0.030513 | CD276 | -1.19 | 0.048316 |
| SWI5 | -1.53 | 0.012367 | TRIM46 | 1.31 | 0.030513 | LINGO2 | -1.19 | 0.048316 |
| NFU1 | 1.62 | 0.012652 | DDRGK1 | -1.31 | 0.030513 | ENTPD2 | 1.19 | 0.048317 |
| ZNF200 | 1.44 | 0.012756 | HCFC1R1 | -1.31 | 0.030513 | DNAH10 | -1.35 | 0.048479 |
| NACC1 | 1.44 | 0.012757 | FOXN1 | 1.31 | 0.030514 | ALAS1 | 1.26 | 0.048792 |
| PDLIM1 | 1.44 | 0.012757 | MRPL1 | 1.31 | 0.030514 | RNGTT | -1.26 | 0.048792 |
| STAMBP | 1.44 | 0.012757 | HDC | 1.24 | 0.030592 | ADCK3 | -1.26 | 0.048792 |
| CCDC152 | 1.44 | 0.012757 | TMEM132D | 1.24 | 0.030592 | ALG8 | 1.12 | 0.049659 |
| ELOVL6 | 1.44 | 0.012757 | COL4A5 | -1.24 | 0.030592 | TMEM200A | 1.12 | 0.049659 |
| FAM216A | -1.44 | 0.012757 | ME2 | 1.24 | 0.030593 | CPNE6 | 1.12 | 0.049659 |
| MTMR4 | -1.44 | 0.012757 | RHOB | 1.24 | 0.030593 | MDH1 | 1.12 | 0.049659 |
| THNSL1 | -1.52 | 0.012903 | KLC2 | 1.24 | 0.030593 | GBP5 | 1.12 | 0.049659 |
| NEDD1 | -1.52 | 0.012903 | BRAP | 1.24 | 0.030593 | SLC36A2 | -1.12 | 0.049659 |
| CSNK1E | 1.52 | 0.012904 | C5orf64 | 1.24 | 0.030593 | CXorf67 | -1.12 | 0.049659 |
| BDNF | 1.52 | 0.012904 | OR2T4 | 1.24 | 0.030593 | IFNA4 | 1.12 | 0.04966 |
| AP1S3 | 1.52 | 0.012904 | CCDC177 | 1.24 | 0.030593 | LPGAT1 | 1.12 | 0.04966 |
| LOC643802 | -1.52 | 0.012904 | SOX9 | 1.24 | 0.030593 | MAGEB3 | 1.12 | 0.04966 |
| MNX1 | -1.52 | 0.012904 | NUP210L | 1.24 | 0.030593 | WFDC10B | 1.12 | 0.04966 |
| TMEM187 | -1.52 | 0.012904 | MVB12B | 1.24 | 0.030593 | ISL1 | 1.12 | 0.04966 |
| SFPQ | 1.61 | 0.013182 | VDAC2 | 1.24 | 0.030593 | ACOT9 | 1.12 | 0.04966 |
| PSTPIP2 | 1.43 | 0.013359 | PURA | 1.24 | 0.030593 | C11orf21 | 1.12 | 0.04966 |
| DAZ2 | 1.43 | 0.013359 | ATL2 | 1.24 | 0.030593 | VAT1 | 1.12 | 0.04966 |
| CHST7 | 1.43 | 0.013359 | ACR | 1.24 | 0.030593 | INSRR | 1.12 | 0.04966 |
| DNAJC25 | 1.43 | 0.013359 | OS9 | 1.24 | 0.030593 | HLA-DRA | 1.12 | 0.04966 |
| SPIN1 | 1.43 | 0.013359 | AAMDC | -1.24 | 0.030593 | DTX3L | -1.12 | 0.04966 |
| GLB1 | -1.43 | 0.013359 | SKA2 | -1.24 | 0.030593 | TAS2R7 | -1.12 | 0.04966 |
| MGP | -1.43 | 0.013359 | CCR5 | -1.24 | 0.030593 | SORL1 | -1.12 | 0.04966 |
| ZNF709 | 1.43 | 0.01336 | TOP1 | -1.24 | 0.030593 | SEZ6L2 | -1.12 | 0.04966 |
| DAZ1 | 1.43 | 0.01336 | BUD13 | -1.24 | 0.030593 | KIFC2 | -1.12 | 0.04966 |
| ATG4A | 1.43 | 0.01336 | ABCA13 | -1.24 | 0.030593 | OLR1 | -1.12 | 0.04966 |
| DAZ4 | 1.43 | 0.01336 | GLYATL3 | -1.24 | 0.030593 | PTPRF | -1.12 | 0.04966 |
| MANBA | -1.43 | 0.01336 | CAV3 | -1.24 | 0.030593 | BDH1 | -1.12 | 0.04966 |
| IKZF2 | -1.43 | 0.01336 | LIPF | -1.24 | 0.030593 | LONP1 | 1.18 | 0.04972 |
| CD1C | -1.43 | 0.01336 | SASS6 | 1.39 | 0.030681 | ALAD | 1.18 | 0.04979 |
| ZSWIM5 | -1.43 | 0.01336 | NDUFA11 | 1.3 | 0.031743 | FRG2 | -1.18 | 0.04981 |
| HGD | -1.43 | 0.01336 | RAD23B | 1.3 | 0.031743 | MRPL44 | 1.18 | 0.04988 |
| CDK13 | 1.51 | 0.013466 | TMA16 | 1.3 | 0.031743 | PRSS56 | 1.18 | 0.04994 |
| TBRG4 | 1.6 | 0.013733 | GDPD5 | 1.3 | 0.031743 |  |  |  |
| REEP5 | 1.42 | 0.013997 | RABGEF1 | -1.3 | 0.031743 |  |  |  |
| OR5AK2 | 1.42 | 0.013997 | CPSF1 | -1.38 | 0.031816 |  |  |  |
| CLEC5A | 1.42 | 0.013997 | ARL2 | -1.38 | 0.031816 |  |  |  |
| PKHD1 | 1.42 | 0.013997 | DAZ3 | 1.23 | 0.031874 |  |  |  |

List of genes and their corresponding RANKS score affecting the growth of NALM-6 cells in the presence of 1C8. Only statistically significant genes (*p* < 0.05) are shown. Note that SRSF10 is essential in NALM-6 cells, as are SRSF1/2/3/7/11.
